# First Isolation, Genomic Characterization and Experimental Infection of Bovine Coronavirus from Sheep

**DOI:** 10.3390/ani16203276

**Published:** 2026-10-17

**Authors:** Xiji Ye, Yurong Su, Cheng Tang, Wujiamu Ouzhi, Yunxin Ren, Can Bao, Yuxi Zhao, Wangqing Banma, Bin Zhang, Hua Yue, Xi Chen

**Affiliations:** 1 Key Laboratory of Qinghai-Tibetan Plateau Animal Genetic Resource Reservation and Utilization, Southwest Minzu University, Ministry of Education, Chengdu 610041, China; yeshekyid@163.com (X.Y.); tangcheng101@163.com (C.T.); ouzhiwujiamu@stu.sicau.edu.cn (W.O.); 200906022015@stu.swun.edu.cn (Y.R.); baocan0000@163.com (C.B.); binovy@sina.com (B.Z.); yhua900@163.com (H.Y.); 2 Agriculture and Rural Affairs Bureau of Ebian Yi Autonomous County, Leshan 614300, China; su15883477140@outlook.com; 3 Institute of Animal Science, Ningxia Academy of Agriculture and Forestry Sciences, Yinchuan 750002, China; zyxwpc18214@gmail.com; 4 Animal Disease Prevention and Control Center of Aba Prefecture, Barkam 624000, China; 17345528071@163.com

**Keywords:** bovine coronavirus, sheep, virus isolation, genome characterization, phylogenetic analysis

## Abstract

Bovine coronavirus (BCoV) is an important pathogen of cattle and has also been detected in several non-bovine ruminants. Although BCoV infection has been reported in sheep, virus isolation and genomic characterization from this host have not been described. In this study, two BCoV isolates were successfully recovered from fecal samples of sheep during an outbreak involving enteric and respiratory signs in Qinghai Province, China. Nearly complete genomes of the two isolates were obtained and were 100% identical to each other across the complete coding regions. Comparative genomic analysis showed that the complete coding regions of the sheep-derived isolates shared 100% nucleotide identity with a previously reported yak-derived BCoV strain, Yak/SWUN/NF2/2022 (OR603986). Phylogenetic analyses based on complete coding regions, *S* gene, and *HE* gene sequences showed that the sheep-derived isolates clustered with yak-derived BCoV strains previously identified in Qinghai Province, suggesting that closely related BCoV lineages may circulate among different ruminant hosts in the Qinghai–Tibet Plateau. Experimental infection provided evidence that the representative sheep-derived isolate was capable of establishing infection in sheep, as evidenced by viral RNA detection, seroconversion, and virus re-isolation from lung tissue. To our knowledge, this study represents the first successful isolation and genomic characterization of BCoV from sheep and provides new insights into the host range, genetic diversity, and potential distribution of closely related BCoV lineages among high-altitude ruminants.

## Simple Summary

Bovine coronavirus is an important cause of intestinal and respiratory disease in cattle, but its occurrence in sheep is poorly understood. In this study, fecal samples were collected from a sheep flock experiencing diarrhea and respiratory signs in Qinghai Province, China. Two bovine coronavirus strains were successfully isolated from affected sheep, and their genomes were analyzed. The complete coding regions of the two sheep-derived BCoV isolates shared 100% nucleotide identity with a previously reported yak-derived BCoV strain from Qinghai Province, indicating a close genetic relationship between BCoV strains detected in sheep and yak. In a preliminary infection experiment, the virus established infection in sheep and caused mild respiratory signs. Viral RNA was detected in respiratory and intestinal tissues, antibody responses were induced, and infectious virus was reisolated from lung tissue. These findings provide evidence that sheep are susceptible to BCoV infection and suggest that closely related BCoV lineages may circulate among different livestock species on the Qinghai–Tibet Plateau. Continued surveillance of livestock sharing grazing environments may help clarify the epidemiology and potential interspecies transmission patterns of BCoV.

## 1. Introduction

Bovine coronavirus (BCoV), a member of the genus Betacoronavirus within the family Coronaviridae, is an important pneumoenteric pathogen of cattle and is associated with neonatal calf diarrhea, winter dysentery in adult cattle, and respiratory disease in cattle of different ages [1,2]. The BCoV genome encodes five major structural proteins, including hemagglutinin-esterase (HE), spike (S), envelope (E), membrane (M), and nucleocapsid (N) proteins [3]. Among these proteins, the S protein mediates receptor recognition and membrane fusion and represents a major target of neutralizing antibodies [4,5,6], whereas HE possesses receptor-binding and receptor-destroying activities that may facilitate viral attachment, release, and spread [7,8]. Genetic variation in these proteins has been associated with changes in antigenicity, tissue tropism, and host adaptation in coronaviruses [4,9].

BCoV has a broad host range and has been detected in numerous domestic and wild ruminants, including giraffes, water deer, white-tailed deer, antelopes, waterbuck, sheep, and goats [10]. More recently, experimental studies have demonstrated that BCoV is capable of infecting mice, suggesting a broader potential host range beyond ruminants [11,12]. BCoV-related strains detected in non-bovine hosts generally show high genetic similarity to bovine-derived strains and lack well-defined host-specific molecular markers, suggesting that these viruses may circulate across different ruminant hosts [13]. Therefore, comparative analysis of BCoV strains from different hosts and geographic regions is important for understanding its genetic diversity, evolutionary relationships, and interspecies transmission potential.

Evidence of BCoV infection in small ruminants has accumulated over the past several decades. Early serological investigations demonstrated exposure to BCoV in both goats and sheep, with antibody prevalence varying among geographic regions [14,15,16]. More recently, molecular studies have detected BCoV RNA in diarrheic goats at relatively high frequencies, suggesting that active infection may be more common than previously recognized [17]. In addition, the successful isolation and genomic characterization of BCoV from goats with acute interstitial pneumonia in 2023 provided the first genome-level evidence of BCoV circulation in small ruminants [18]. More recently, a novel Betacoronavirus 1, designated caprine coronavirus (cpCoV), was identified in goats with diarrhea in China, further expanding our understanding of coronavirus diversity in ruminant hosts [19]. Collectively, these findings indicate that coronaviruses related to BCoV are widely distributed among small-ruminant populations. However, despite previous evidence suggesting BCoV exposure or coronavirus-associated infection in sheep, successful virus isolation and genomic characterization of sheep-derived BCoV strains have not been reported.

In March 2023, respiratory and enteric disease was observed in a sheep flock in Qinghai Province, China, and BCoV RNA was detected in fecal samples from affected animals. Given the limited information available on sheep-derived BCoV, the present study was designed to isolate infectious virus from clinical samples, characterize the genomes of the obtained isolates, and assess their genetic relationships with BCoV strains from other hosts. In addition, a small-scale experimental infection study was performed to preliminarily evaluate whether a representative sheep-derived isolate could establish infection in sheep. These data provide new evidence for BCoV circulation in sheep and contribute to a better understanding of its host range and potential interspecies transmission among ruminants.

## 2. Materials and Methods

### 2.1. Clinical Sample Collection

In March 2023, an outbreak of respiratory and enteric disease characterized by cough, serous-to-mucoid nasal discharge, and diarrhea occurred on a sheep farm in Qinghai Province, China (approximately 3000 m above sea level). The flock consisted of 514 sheep aged 2 months to 4 years, of which 87 animals developed clinical signs during the two-month outbreak period, corresponding to a cumulative morbidity of approximately 16.9%. No mortality was observed during the outbreak.

For pathogen screening, 14 fecal samples were collected from diseased sheep and 41 from clinically healthy sheep in the same flock. Fresh fecal samples were collected directly from the rectum of individual animals using sterile disposable gloves, placed into sterile tubes, transported on ice to the laboratory, and stored at −80 °C until use.

### 2.2. Cells and Experimental Animals

Human ileocecal adenocarcinoma HCT-8 cells (ATCC CCL-244) were maintained in RPMI-1640 medium (Gibco, Grand Island, NY, USA; Cat. No. 11875093) supplemented with 10% fetal bovine serum (FBS; Gibco, Grand Island, NY, USA; Cat. No. A5669701). Cells were cultured at 37 °C in a humidified atmosphere containing 5% CO_2_.

Three-month-old male Tibetan sheep used for experimental infection were purchased from Sichuan Tiandi Sheep Bioengineering Co., Ltd. (Chengdu, China). The animals were clinically healthy and acclimated before inoculation. Animals were housed in separate rooms according to group, with free access to feed and water throughout the study, and were monitored daily for clinical signs and general health status.

### 2.3. Sample Processing and Nucleic Acid Extraction

Approximately 1 g of fecal sample was suspended in 9 mL of phosphate-buffered saline (PBS; Jiangsu Aidisheng Biotechnology Co., Ltd., Yancheng, China; Cat. No. ADS053IH0) to prepare a 10% (*w*/*v*) fecal suspension. The suspension was thoroughly homogenized by vigorous vortexing and clarified by centrifugation at 8000× *g* for 10 min at 4 °C. The resulting supernatant was collected and used for subsequent pathogen detection and virus isolation.

For RNA extraction, 200 μL of supernatant was mixed with 500 μL of TRIzol reagent (Invitrogen, Carlsbad, CA, USA; Cat. No. 15596026CN), vortexed, and incubated at room temperature for 15 min. After adding 200 μL of chloroform, the mixture was shaken vigorously, incubated for 5 min at room temperature, and centrifuged at 12,000× *g* for 15 min at 4 °C. The upper aqueous phase was transferred to an RNase-free tube and mixed with 250 μL of isopropanol. Following incubation for 10 min at room temperature, RNA was pelleted at 12,000× *g* for 10 min at 4 °C. The pellet was washed with 1 mL of 75% ethanol and centrifuged at 7500× *g* for 10 min at 4 °C, air-dried for 5–10 min, and dissolved in 20 μL of RNase-free water.

For DNA extraction, 300 μL of supernatant was mixed with 500 μL of STE buffer, 20 μL of proteinase K (10 mg/mL), and 20 μL of SDS (10%), and incubated at 57 °C for 2 h. The lysate was mixed with 500 μL of phenol:chloroform alcohol (25:24:1, *v*/*v*/*v*; Sigma-Aldrich, St. Louis, MO, USA; Cat. No. P3803) and centrifuged at 10,000 rpm for 5 min. Subsequently, 500 μL of the upper aqueous phase was extracted with an equal volume of chloroform alcohol (24:1, *v*/*v*) and centrifuged at 10,000 rpm for 3 min. A 300-μL aliquot of the aqueous phase was mixed with 30 μL of 5 mol/L NaCl and 1 mL of absolute ethanol, incubated at 4 °C for 15 min, and centrifuged at 10,000 rpm for 3 min. The DNA pellet was washed with 500 μL of 70% ethanol and centrifuged at 10,000 rpm for 2 min, air-dried, and dissolved in 20 μL of double-distilled water.

RNA and DNA concentrations were determined spectrophotometrically based on absorbance at 260 nm, and purity was evaluated using the A260/A280 absorbance ratio. The purified nucleic acids were stored at −80 °C until further analysis.

### 2.4. Detection of Common Pathogens

BCoV RNA was detected using a two-step TaqMan RT-qPCR assay targeting the nucleocapsid (*N*) gene, which was established in the present study. For reverse transcription, total RNA was converted into cDNA using the HiScript III 1st Strand cDNA Synthesis Kit (Vazyme Biotech Co., Ltd., Nanjing, China; Cat. No. R312-01) according to the manufacturer’s protocol. The cDNA was then used as the template for quantitative PCR.

To design specific primers and a probe, a total of 320 BCoV *N* gene reference sequences available in GenBank as of January 2025 were aligned using MegAlign (DNASTAR Inc., Madison, WI, USA; Lasergene version 7.1). Conserved regions were identified based on the sequence alignment, and candidate primers and probes were designed using Primer Premier 5.0 (PREMIER Biosoft International, Palo Alto, CA, USA). The final primer–probe combination was selected to target a highly conserved region of the BCoV *N* gene, with the aim of minimizing sequence mismatches among the reference strains.

The primers and probe targeting BCoV were as follows: F, 5′-CGTCAATTGCTGCCACGAT-3′; R, 5′-CGTCAATGTCGGTGCCATAC-3′; and probe, 5′-VIC-CTTGGAACAGGACCGCATGCCA-TAMRA-3′. Each qPCR reaction was performed in a final volume of 20 μL, containing 10 μL of Premix Ex Taq (Probe qPCR; Takara, Shiga, Japan; Cat. No. RR390A), 0.8 μL of forward primer (20 μM), 0.8 μL of reverse primer (20 μM), 0.4 μL of probe (10 μM), 2 μL of cDNA template, and 6 μL of nuclease-free water. Amplification was conducted under the following conditions: initial denaturation at 95 °C for 3 min, followed by 40 cycles of 95 °C for 15 s and 56 °C for 30 s. A recombinant plasmid containing the BCoV *N* gene target fragment was used as the positive control, while a negative extraction control and a no-template control (nuclease-free water) were included to monitor potential contamination. Samples showing a typical amplification curve with a Ct value < 40 were regarded as BCoV-positive. The analytical performance of the established assay was evaluated by determining analytical specificity, limit of detection (LOD), and amplification efficiency. The analytical specificity was evaluated using nucleic acids positive for BCoV, bovine rotavirus group A (BRVA), bovine viral diarrhea virus (BVDV), infectious bovine rhinotracheitis virus (IBRV), bovine norovirus (BNoV), bovine parvovirus (BPV), bovine torovirus (BToV), nebovirus (NeV), bovine kobuvirus (BKoV), *Salmonella enterica*, and *enterotoxigenic Escherichia coli* (ETEC).

To investigate potential co-infections with other enteric pathogens, the clinical fecal samples were also screened for BRVA, BVDV, enterovirus, sheep aichivirus D, *Salmonella* spp., ETEC, Pasteurellaceae, and *Cryptosporidium* spp. The corresponding primer sequences and amplification conditions used for pathogen screening are provided in Supplementary Table S1. The analytical sensitivity was determined using 10-fold serial dilutions of a recombinant plasmid containing the BCoV *N* gene target fragment. The assay showed a detection limit of 10 copies per reaction, with a standard curve of Ct = −3.21 × log_10_[copies] + 39.286 (R^2^ = 0.999) and an amplification efficiency of 104.9%.

Given the limited sample size, BCoV detection rates in diarrheic and clinically healthy sheep were compared using Fisher’s exact test. Statistical analyses were performed using SPSS version 27.0 (IBM Corp., Armonk, NY, USA), and *p* < 0.05 was considered statistically significant.

### 2.5. Virus Isolation and Plaque Purification

Virus isolation was attempted using HCT-8 cells from ten BCoV-positive fecal suspensions with low Ct values. Before inoculation, the clarified fecal supernatants were passed through 0.22-μm syringe filters (Biosharp, Beijing, China; Cat. No. BS-PVDF13-22-B) and supplemented with penicillin–streptomycin solution (Gibco, USA; Cat. No. 15140-122) to final concentrations of 100 U/mL penicillin and 100 μg/mL streptomycin. The prepared inocula were added to HCT-8 cell monolayers at approximately 80% confluence and allowed to adsorb for 1 h at 37 °C in a 5% CO_2_ incubator. The inocula were then removed, and the cells were rinsed three times with phosphate-buffered saline (PBS; Jiangsu Aidisheng Biotechnology Co., Ltd., Yancheng, China; Cat. No. ADS053IH0) before fresh maintenance medium containing 2% fetal bovine serum (FBS; Gibco, USA; Cat. No. A5669701) was added.

The inoculated cultures were inspected daily for cytopathic effects (CPE). When CPE became evident, the culture supernatant together with infected cells was collected, frozen and thawed three times, and clarified by centrifugation. The clarified lysate was then used to inoculate fresh HCT-8 cells for the next passage. Each sample was subjected to a maximum of three passages. A sample was considered successfully isolated when reproducible CPE was observed during serial passage and BCoV RNA was repeatedly detected in the corresponding cell culture by RT-qPCR.

RT-qPCR-positive cultures were further purified by plaque selection. Briefly, virus-containing supernatants were serially diluted 10-fold and inoculated onto HCT-8 cell monolayers. After viral adsorption, the cells were covered with semisolid overlay medium consisting of maintenance medium supplemented with 1% low-melting-point agarose (Invitrogen, USA; Cat. No. 16520050). When well-separated plaques appeared, individual plaques were picked, resuspended in maintenance medium, and amplified in fresh HCT-8 cells.

The infectivity of the purified isolates was measured by the median tissue culture infectious dose assay (TCID_50_) in HCT-8 cells. Serial 10-fold dilutions of virus stocks were added to replicate wells, and cultures were monitored for CPE. Viral titers were calculated using the Reed–Muench method and expressed as TCID_50_/mL. The general procedures for virus isolation, plaque purification, and virus titration were based on our laboratory’s previously described virological workflow with modifications [20].

### 2.6. Identification of Viral Isolates

#### 2.6.1. RT-qPCR and Sequencing Confirmation

Following plaque purification, the purified isolates were further tested using the BCoV TaqMan RT-qPCR assay described in Section 2.4. Selected amplification products were subjected to Sanger sequencing by Sangon Biotech Co., Ltd. (Shanghai, China). The resulting sequences were compared with reference sequences in the NCBI database using BLASTn (BLAST+ version 2.17.0, NCBI, Bethesda, MD, USA) to confirm viral identity.

#### 2.6.2. Indirect Immunofluorescence Assay (IFA)

The antigenicity of the purified isolates was examined by indirect immunofluorescence assay. HCT-8 cells grown in 24-well plates were inoculated with purified BCoV isolates and incubated until BCoV-associated CPE was observed. The cells were then fixed with pre-cooled 80% acetone (Kermel, Tianjin, China; Cat. No. KM20218) at −20 °C for 20 min and air-dried. After blocking with 3% BSA in PBS (Solarbio, Beijing, China; Cat. No. A8010) for 1 h, the cells were incubated with rabbit anti-BCoV S2 polyclonal serum prepared in our laboratory at a dilution of 1:500 for 1 h. After washing, Alexa Fluor 568-conjugated goat anti-rabbit IgG (Invitrogen, Carlsbad, CA, USA; Cat. No. A11011) was added as the secondary antibody at a dilution of 1:3000 for 1 h. Cell nuclei were counterstained with DAPI (Biosharp, China; Cat. No. BL105A; diluted 1:2000 in PBS) for 10 min. Mock-infected HCT-8 cells were included as negative controls. Fluorescence was examined using an inverted fluorescence microscope (Olympus, Tokyo, Japan).

#### 2.6.3. Transmission Electron Microscopy (TEM)

When typical CPE was observed in HCT-8 cells infected with the purified BCoV isolate, the culture supernatant was discarded and the cell monolayers were washed several times with PBS. The infected cells, together with cell-associated debris, were collected into sterile tubes and submitted to Lilai Biotechnology Co., Ltd. (Chengdu, China) for ultrastructural examination. The prepared sections were examined using a JEM-1400Flash transmission electron microscope (JEOL, Tokyo, Japan), and images were recorded to evaluate intracellular coronavirus-like particles and associated cellular changes.

### 2.7. Metagenomic Sequencing and Validation

Purified virus-containing culture supernatants were submitted to Sangon Biotech Co., Ltd. (Shanghai, China) for metagenomic sequencing and bioinformatic analysis. Briefly, viral culture supernatants were prepared from BCoV-positive HCT-8 cell cultures showing stable CPE and were shipped on dry ice to the service provider. Nucleic acid pretreatment, RNA extraction, library preparation, high-throughput sequencing, quality control, de novo assembly, and preliminary taxonomic annotation were performed by Sangon Biotech according to its standard metagenomic sequencing workflow. Sequencing was conducted on an Illumina platform. The assembled contigs identified as BCoV-related sequences were retrieved and used for downstream genome comparison and phylogenetic analysis.

To validate the assembled genome sequences, ten pairs of primers (Supplementary Table S2) were designed to amplify overlapping fragments covering the near-complete BCoV genome. PCR products were examined by agarose gel electrophoresis, purified using a PCR purification kit (Tiangen Biotech Co., Ltd., Beijing, China; Cat. No. DP214-02), cloned into the pMD19-T vector (Takara, Japan; Cat. No. 6013), and subjected to Sanger sequencing by Chengdu Qinke Biotechnology Co., Ltd. (Chengdu, China). The Sanger-derived sequences were aligned with the metagenomic assemblies using SeqMan software in the DNASTAR Lasergene package version 7.1 (DNASTAR Inc., Madison, WI, USA). Overlapping regions were manually inspected, and the validated fragments were used to confirm the final near-complete genome sequences.

### 2.8. Comparative Genomic, Phylogenetic, and Recombination Analyses

To determine the evolutionary relationships of the sheep-derived BCoV isolates, all available BCoV genome and gene sequences deposited in GenBank before January 2025 were retrieved. Sequences that were incomplete, incorrectly annotated, or unrelated to BCoV were excluded. The final dataset used for comparative genomic analysis included 280 complete coding regions, 507 complete *S* gene sequences, and 371 complete *HE* gene sequences, including the two sheep-derived BCoV isolates identified in this study. The accession numbers and related information of the reference sequences used in this study are provided in Supplementary Table S3, and the corresponding sequence datasets used for comparative analyses are available in Supplementary Dataset S1–S3. Multiple sequence alignments were generated using ClustalW in MEGA version 7.0. Pairwise nucleotide and deduced amino acid identities between the sheep-derived isolates and reference strains were calculated using MegAlign in the DNASTAR Lasergene package version 7.1.

For phylogenetic analysis, redundant sequences were removed from the 280 complete coding region sequences to avoid overrepresentation of highly similar strains. A representative dataset consisting of 74 complete coding region sequences was generated, and the corresponding complete *S* and *HE* gene sequences from these representative genomes were used for gene-specific phylogenetic analyses. The corresponding sequence datasets used for phylogenetic analyses are available in Supplementary Dataset S4–S6. Maximum-likelihood phylogenetic trees were constructed separately based on the complete coding regions, *S* gene, and *HE* gene sequences using MEGA version 7.0. The most appropriate nucleotide substitution model for each dataset was selected in MEGA, and branch support was evaluated with 1000 bootstrap replicates. Bootstrap values above 60% were displayed on the phylogenetic trees.

To examine whether recombination contributed to the genomic characteristics of the sheep-derived isolates, potential recombination events were screened using RDP version 4.101. Seven algorithms implemented in RDP, including RDP, GENECONV, BootScan, MaxChi, Chimaera, SiScan, and 3Seq, were used for the analysis. Potential recombination events were considered reliable when detected by multiple algorithms with statistically significant support.

### 2.9. Experimental Infection of Sheep with a Representative Isolate

A small-scale animal experiment was performed to preliminarily assess whether the sheep-derived BCoV isolate could establish infection in sheep. Five clinically healthy three-month-old male Tibetan sheep were used. Before inoculation, nasal and rectal swabs from all animals were confirmed to be negative for BCoV RNA by RT-qPCR, and serum samples were negative for BCoV-specific antibodies by ELISA. Three sheep were assigned to the inoculated group, and two sheep served as mock-inoculated controls.

BCoV-specific serum antibodies were detected using an indirect ELISA established in our laboratory. The assay was optimized using recombinant BCoV S2 protein as the coating antigen, and the optimal antigen concentration, serum dilution, and secondary antibody dilution were determined by checkerboard titration. The specificity, sensitivity, and repeatability of the established ELISA were evaluated before application to experimental infection samples. The assay only showed specific reactivity with BCoV-positive sera. The intra- and inter-assay coefficients of variation were within acceptable ranges, demonstrating good repeatability and stability. The positive-to-negative (P/N) ratio was calculated based on the optimized assay conditions, and a P/N ratio > 2.1 was defined as positive according to the established cutoff value. Briefly, 96-well microplates were coated with recombinant BCoV S2 protein (0.1 μg/mL, 100 μL/well) at 37 °C for 1 h. After washing with PBST, the plates were blocked with 2% BSA at 37 °C for 1.5 h. Serially diluted sheep sera were added in duplicate and incubated at 37 °C for 1 h. Following washing, rabbit anti-sheep IgG H&L (HRP) (Abcam, Cambridge, UK; Cat. No. ab97130) was added at a dilution of 1:2500 and incubated at 37 °C for 1 h. After washing, TMB substrate was added for color development, and absorbance was measured at 450 nm after stopping the reaction. A positive-to-negative (P/N) ratio > 2.1 was considered positive, and the endpoint antibody titer was defined as the highest serum dilution yielding a positive result.

Sheep in the experimental group were inoculated simultaneously via the intranasal and oral routes with the third-passage plaque-purified Ovine/SWUN/FA/2023 isolate. A total of 3 mL and 5 mL of virus suspension (10^7^ TCID_50_/mL) was administered intranasally and orally, respectively. The control sheep received the same volumes of RPMI-1640 medium through the corresponding routes. The two groups were housed separately throughout the experiment and were monitored daily for clinical signs, body temperature, and general health status. Serum samples were collected at scheduled time points for serological analysis using the indirect ELISA method described above. Serum samples were collected before inoculation and daily from 1 to 5 DPI. Nasal swabs and fecal samples were collected before inoculation and daily from 1 to 5 DPI to monitor viral shedding. Viral RNA levels were determined using the TaqMan RT-qPCR assay described above. Serially diluted sera were tested in duplicate, and a P/N ratio > 2.1 was considered positive. The endpoint antibody titer was defined as the highest serum dilution yielding a positive result.

At the end of the observation period, all sheep were anesthetized with Zoletil™ 50 (tiletamine–zolazepam; Virbac S.A, Nice, France; Cat. No. 32504) at an approximate dose of 15 mg/kg by intravenous administration. After an adequate depth of anesthesia was confirmed by the absence of response to painful stimuli, the animals were humanely euthanized by carotid artery exsanguination in accordance with the approved animal experimental protocol. Death was confirmed by the absence of heartbeat, respiration, and corneal reflex before necropsy and tissue collection. Samples of the nasal turbinate, trachea, bronchus, lung, duodenum, jejunum, ileum, cecum, colon, rectum, heart, liver, spleen, kidney, and brain were collected for virological and histopathological analyses. Selected tissue samples were used for virus re-isolation. BCoV RNA copy numbers in tissue-derived cDNA were quantified using the TaqMan RT-qPCR assay described above and expressed as viral RNA copies per microliter of cDNA (copies/μL cDNA). Parallel tissue samples were fixed in 4% paraformaldehyde (Alfa Aesar, Haverhill, MA, USA; Cat. No. 49151), embedded in paraffin, sectioned, and stained with hematoxylin and eosin (H&E) for histopathological examination.

For the experimental infection study, tissue viral RNA copy numbers and antibody titers were summarized as mean ± standard deviation (SD). Technical replicates were averaged for each individual animal before group-level calculations. Because of the small number of animals and the descriptive nature of this preliminary infection study, no inferential statistical comparisons were performed. Graphs were generated using GraphPad Prism version 9.0.

## 3. Results

### 3.1. Detection Results for Common Pathogens

BCoV RNA was detected in 13 of 14 fecal samples from sheep with respiratory and enteric signs during the outbreak, corresponding to a positivity rate of 92.9%. In contrast, 20 of 41 fecal samples from clinically healthy sheep in the same flock were positive for BCoV RNA, with a positivity rate of 48.8%. The detection rate of BCoV RNA was significantly higher in diseased sheep than in clinically healthy sheep (*p* = 0.00041, Fisher’s exact test). None of the other tested enteric pathogens were detected in these fecal samples. The Ct values of BCoV-positive samples from diseased and clinically healthy sheep are summarized in Supplementary Table S4. Diseased sheep generally showed lower Ct values than clinically healthy sheep, suggesting relatively higher levels of viral RNA.

### 3.2. Isolation and Identification of BCoV from Sheep Fecal Samples

Ten fecal samples with relatively high BCoV RNA loads were selected for virus recovery in HCT-8 cells. During the first passage, six inoculated cultures showed morphological changes suggestive of CPE. However, after three consecutive passages, only two cultures derived from diarrheic sheep reproducibly developed CPE and remained positive for BCoV RNA by RT-qPCR.

**Figure 1:**
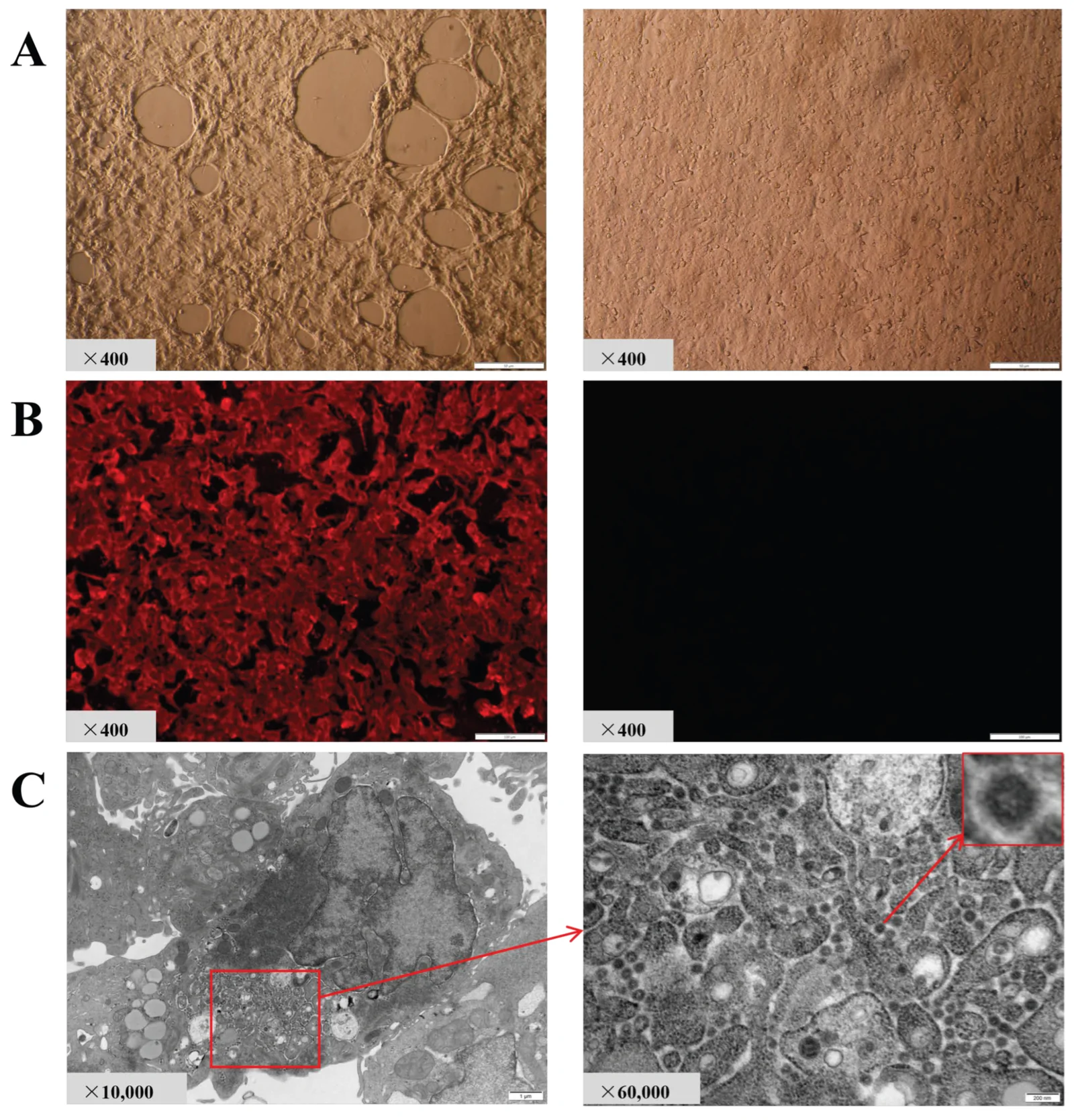
Isolation and identification of sheep-derived BCoV isolates. (**A**) Cytopathic effects observed in HCT-8 cells at 48 h post-inoculation with the purified BCoV isolate, left, infected cells; right, mock-infected cells. (**B**) IFA of infected HCT-8 cells at 24 h post-inoculation, left, infected cells; right, mock-infected cells. (**C**) Ultrathin-section TEM of infected HCT-8 cells showing coronavirus-like particles at ×10,000 and ×60,000 magnification.

Cytopathic changes became visible at approximately 24 h post-inoculation and were characterized by cell shrinkage and variably sized cytoplasmic vacuoles. At 48 h post-inoculation, the affected cells formed a reticular pattern, followed by partial detachment from the culture surface and accumulation of floating cellular debris in the medium (Figure 1A). After plaque purification and propagation to the third passage, the two isolates Ovine/SWUN/FA/2023 and Ovine/SWUN/28-3/2023 retained similar CPE characteristics and reached infectious titers of 10^7.89^ and 10^8.20^ TCID_50_/mL, respectively.

The identity of the two isolates was confirmed by RT-qPCR and indirect immunofluorescence assay using rabbit anti-BCoV S2 polyclonal serum (Figure 1B). Ultrathin-section transmission electron microscopy further revealed coronavirus-like particles with an approximate diameter of 90 nm in infected HCT-8 cells (Figure 1C).

### 3.3. Molecular Characteristics of the Sheep-Derived BCoV Genome

#### 3.3.1. Genome Characterization and Phylogenetic Analysis

Following sequencing, two nearly complete genomes of sheep-derived BCoV were obtained from diarrheic fecal samples and designated Ovine/SWUN/FA/2023 (OR676674) and Ovine/SWUN/28-3/2023 (OR676675). The assembled genome lengths of FA and 28-3 were 31,600 nt and 31,103 nt, respectively. Both genomes contained complete coding regions of 30,528 nt that shared 100% nucleotide identity. Comparison with all 280 complete coding region sequences retrieved from GenBank, including 250 bovine-derived and 30 non-bovine-derived genomes, revealed nucleotide identities of the complete coding regions ranging from 97.5% to 100%. The deduced amino acid identities of the coding regions ranged from 96.9% to 100%.

The sheep-derived isolates showed the highest genomic similarity to the yak-derived strain Yak/SWUN/NF2/2022 (OR603986). The assembled genome lengths of Ovine/SWUN/FA/2023 (OR676674), Ovine/SWUN/28-3/2023 (OR676675), and Yak/SWUN/NF2/2022 (OR603986) were 31,600 nt, 31,103 nt, and 31,108 nt, respectively. Comparison of the three genomes revealed a shared aligned region of 31,103 nt, within which the pairwise nucleotide identities were 99.7621% between FA and 28-3, 99.7428% between FA and NF2, and 99.7557% between 28-3 and NF2. An aligned region of 30,997 nt showed complete nucleotide identity among the three strains, including the complete coding regions (30,528 nt), which showed 100% nucleotide identity. The nucleotide differences were mainly located near the 5′ and 3′ terminal regions, corresponding to variations in terminal sequences and genome assembly boundaries. These results indicate that the sheep-derived isolates and Yak/SWUN/NF2/2022 shared an extremely conserved coding region, while limited sequence differences were present mainly at the genomic termini.

**Figure 2:**
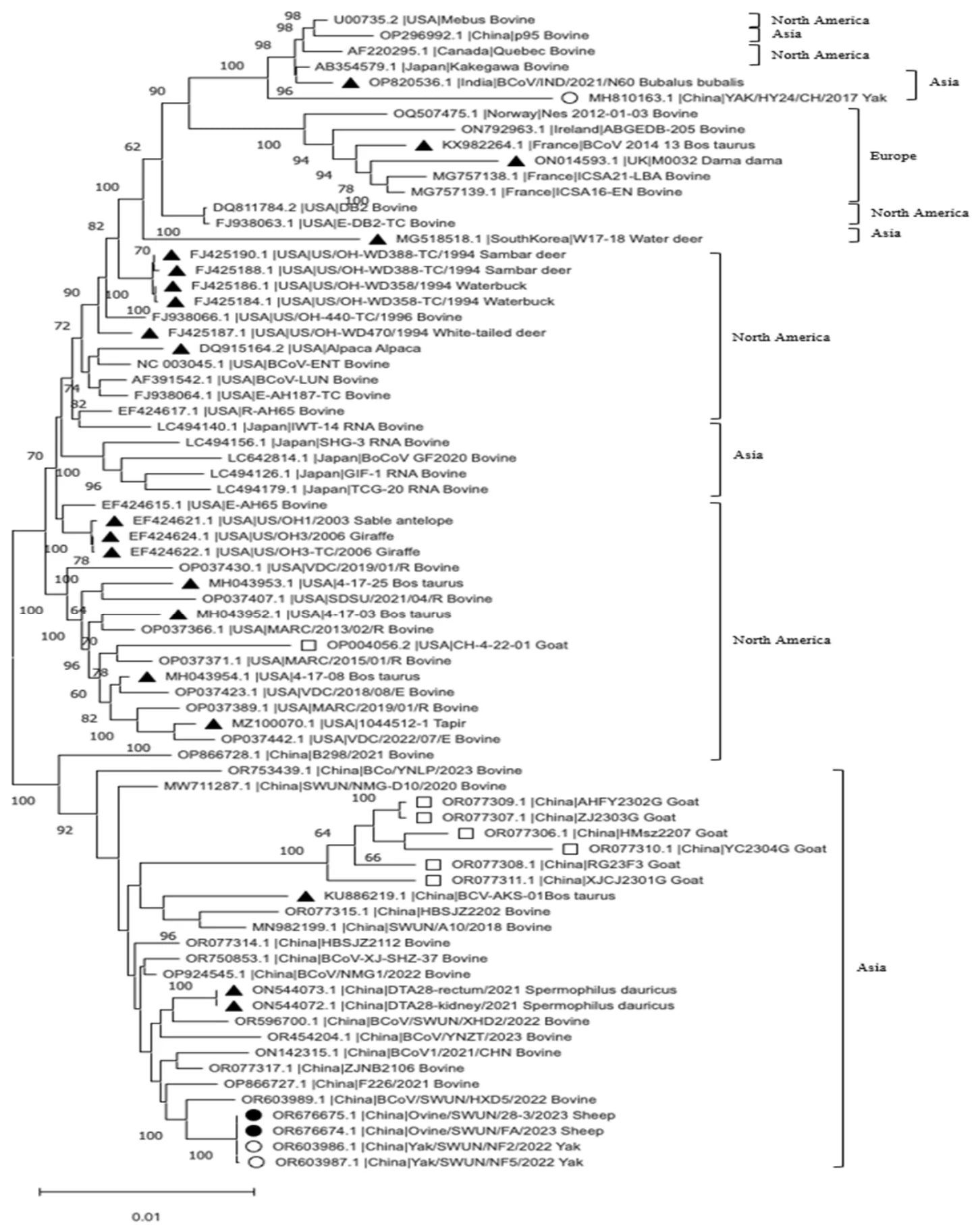
Phylogenetic analysis of representative BCoV genome (complete coding regions). The maximum-likelihood tree was constructed based on representative complete coding region sequences using the GTR + G + I nucleotide substitution model. The dataset included 42 representative bovine-derived BCoV sequences, 30 non-bovine-derived BCoV sequences, and the two sheep-derived isolates identified in this study. The sheep-derived isolates are indicated by solid circles (●). Yak-derived strains are indicated by open circles (○), goat-derived strains by squares (□), and strains from other non-bovine hosts by triangles (▲). Bootstrap values (>60%) are shown at major nodes.

Highly redundant bovine-derived genomes were excluded to reduce overrepresentation of closely related sequences while retaining representative strains from different geographic regions and collection periods. The resulting dataset comprised 42 representative bovine-derived genomes together with all 30 non-bovine-derived genomes. Phylogenetic analysis of this representative dataset produced the same overall topology as the complete dataset, with the two sheep-derived isolates clustering within the major Asian BCoV lineage and forming a distinct sub-clade together with the two yak-derived strains previously identified in Qinghai Province, although they were obtained from different sampling locations (Figure 2). The maximum-likelihood phylogenetic tree constructed using the complete dataset of 280 BCoV complete coding region sequences retrieved from GenBank, together with the two sheep-derived isolates identified in this study, is provided in Supplementary Figure S1.

Recombination analysis using seven algorithms implemented in RDP 4.101, including RDP, GENECONV, BootScan, MaxChi, Chimaera, SiScan, and 3Seq, revealed no detectable recombination events in the sheep-derived BCoV genomes under the predefined criteria.

#### 3.3.2. Molecular Characteristics of the *S* Gene

**Figure 3:**
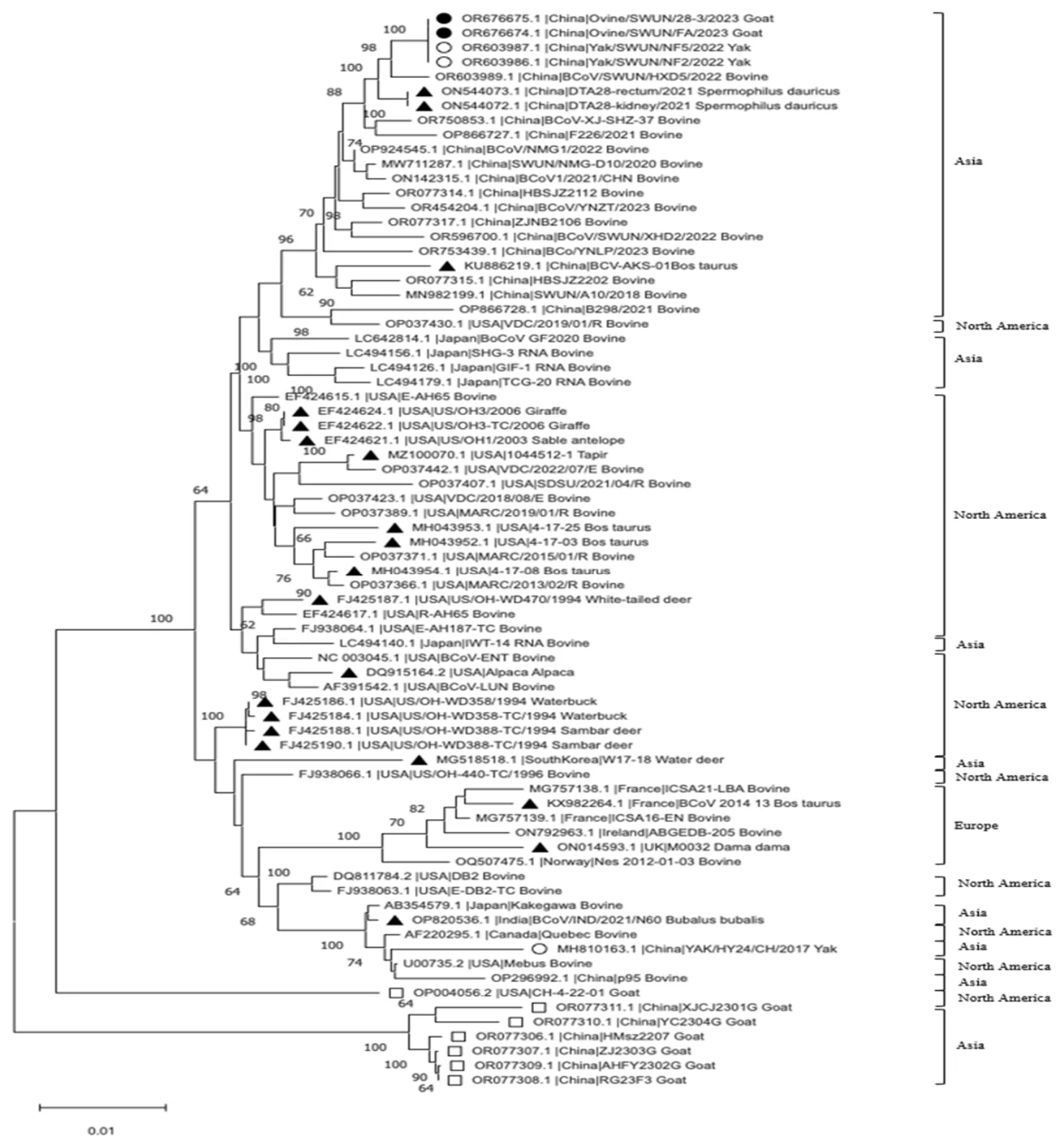
Phylogenetic analysis of representative BCoV *S* gene sequences. The maximum-likelihood tree was constructed based on representative complete *S* gene sequences using the TN93 + G + I nucleotide substitution model. The dataset included 42 representative bovine-derived BCoV *S* gene sequences, 30 non-bovine-derived BCoV *S* gene sequences, and the two sheep-derived isolates identified in this study. The sheep-derived isolates are indicated by solid circles (●). Yak-derived strains are indicated by open circles (○), goat-derived strains by squares (□), and strains from other non-bovine hosts by triangles (▲). Bootstrap values (>60%) are shown at major nodes.

The complete *S* gene of both sheep-derived BCoV isolates was 4092 bp, encoding 1363 amino acids, with 100% nucleotide identity between them. Comparison with all 507 BCoV *S* genes retrieved from GenBank revealed nucleotide identities ranging from 95.9% to 100%. The *S* genes of the sheep-derived isolates were identical to those of the two Qinghai yak-derived strains, Yak/SWUN/NF2/2022 and Yak/SWUN/NF5/2022. Notably, the sheep-derived isolates and the two Qinghai yak-derived strains shared two amino acid substitutions that were not detected in any of the other BCoV sequences included in the present dataset: M118R in the S1 subunit and M44T in the S2 subunit, with positions numbered relative to the corresponding subunits. Phylogenetic analysis showed that *S* genes from non-bovine hosts generally clustered with geographically related bovine-derived strains. In particular, the sheep-derived isolates and yak-derived strains formed a distinct sub-clade, indicating a close evolutionary relationship among these strains (Figure 3).

#### 3.3.3. Molecular Characteristics of the *HE* Gene

**Figure 4:**
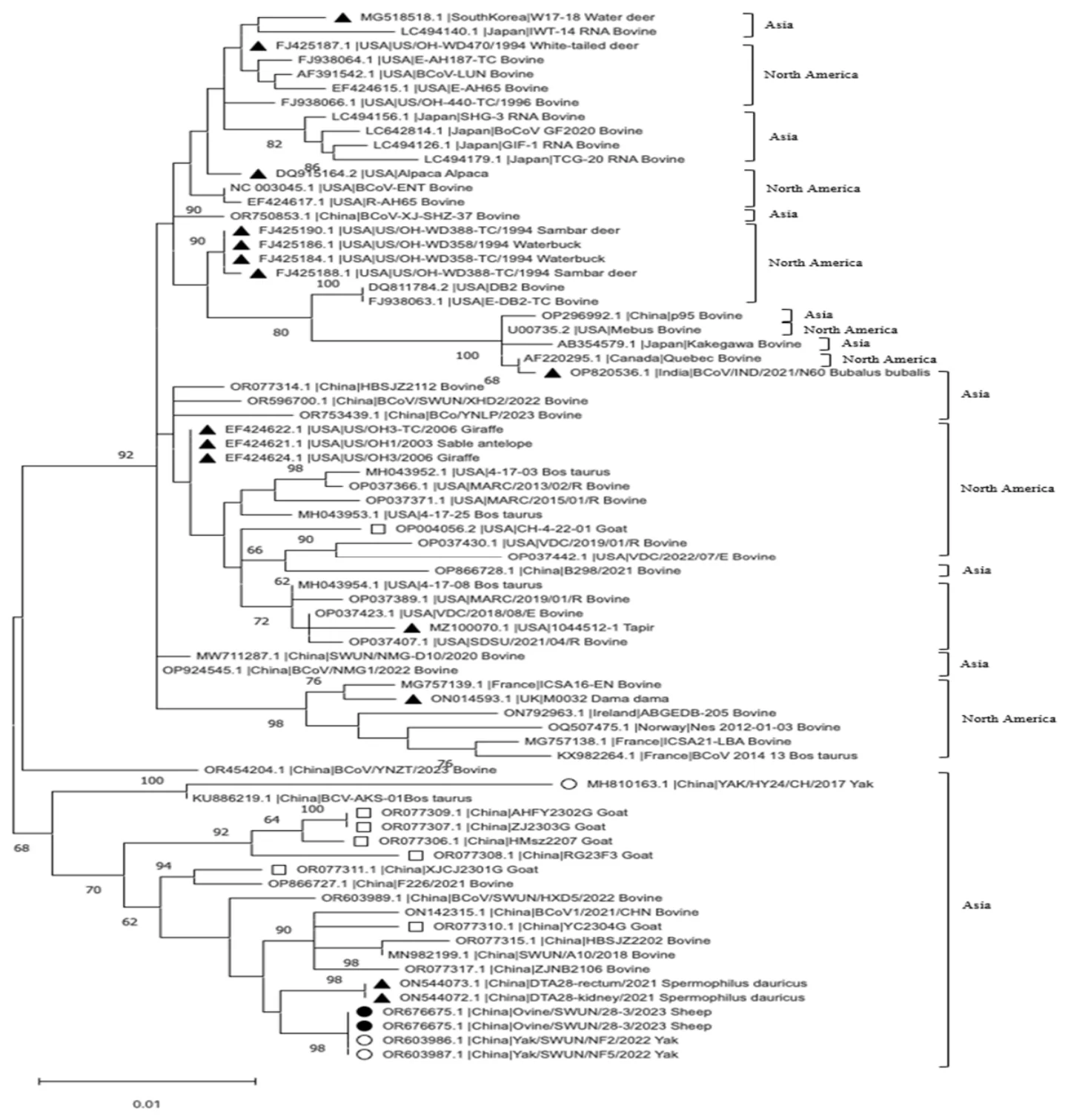
Phylogenetic analysis of representative BCoV *HE* gene sequences. The maximum-likelihood tree was constructed based on representative complete *HE* gene sequences using the TN93 + G + I nucleotide substitution model. The dataset included 42 representative bovine-derived BCoV *HE* gene sequences, 30 non-bovine-derived BCoV *HE* gene sequences, and the two sheep-derived isolates identified in this study. The sheep-derived isolates are indicated by solid circles (●). Yak-derived strains are indicated by open circles (○), goat-derived strains by squares (□), and strains from other non-bovine hosts by triangles (▲). Bootstrap values (>60%) are shown at major nodes.

The complete *HE* gene of both sheep-derived BCoV isolates was 1275 bp, encoding 424 amino acids, with 100% nucleotide identity between them. Comparison with all 393 BCoV *HE* genes retrieved from GenBank revealed nucleotide identities ranging from 96.0% to 100%. The *HE* genes of the sheep-derived isolates were identical to those of the two yak-derived strains previously identified in Qinghai Province, Yak/SWUN/NF2/2022 and Yak/SWUN/NF5/2022. Notably, the sheep-derived isolates and the two Qinghai yak-derived strains shared an HE amino acid substitution, P384S, which was not detected in the other Asian BCoV sequences included in the present dataset. Phylogenetic analysis showed that *HE* genes from non-bovine hosts generally clustered with geographically related bovine-derived strains. In particular, the sheep-derived isolates and yak-derived strains formed a distinct sub-clade, further supporting their close regional genetic relationship (Figure 4).

### 3.4. Experimental Infection of Sheep with the Representative Isolate

**Figure 5:**
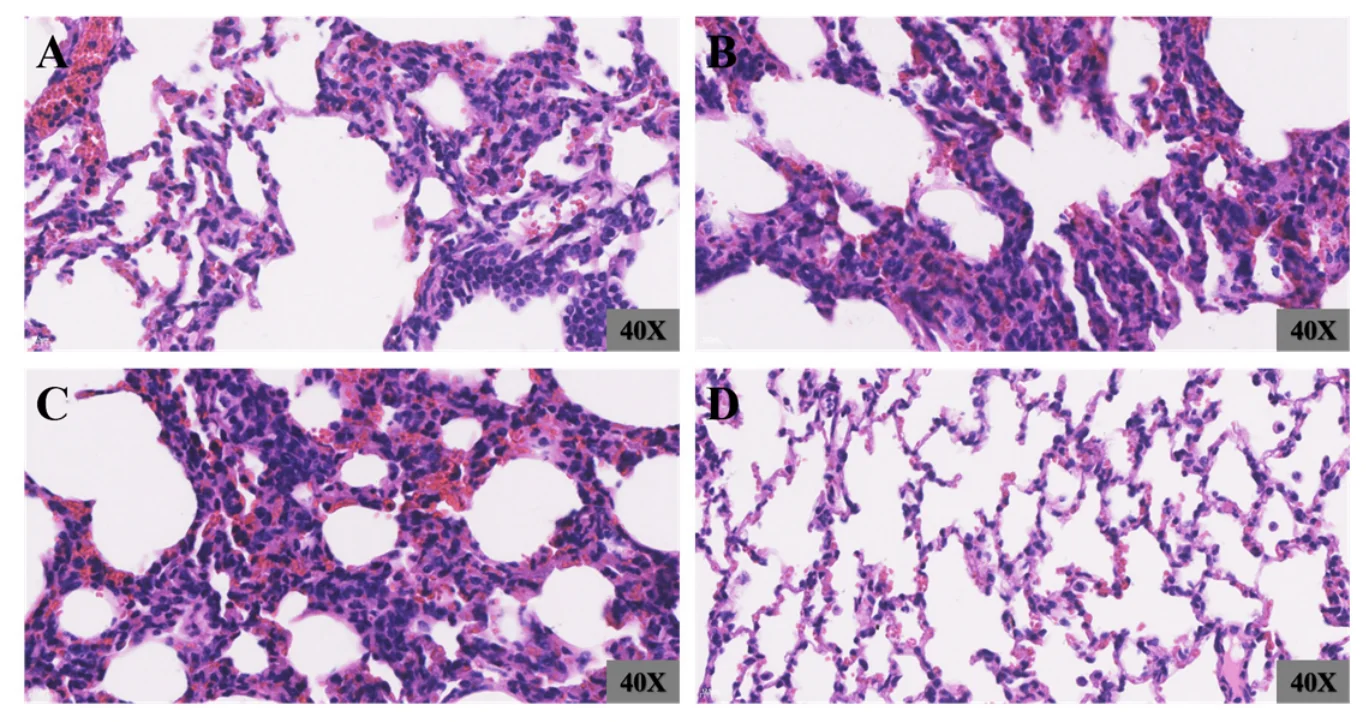
Histopathological changes in lung tissues of sheep experimentally infected with sheep-derived BCoV. (**A**–**C**) Lung tissues from experimentally infected sheep showing widening of the alveolar septa and thickened alveolar walls, vascular congestion and dilation, interstitial pulmonary edema with mild mononuclear inflammatory cell infiltration, mild alveolar structural disturbance or focal alveolar collapse, and focal hemorrhage or erythrocyte extravasation. (**D**) Representative lung tissue from a control sheep showing normal histological architecture. H&E staining; scale bars = 200 μm.

Following experimental infection, infected sheep showed reduced feed intake, mild elevations in body temperature, and serous-to-mucoid nasal discharge during the 5-day observation period. No diarrhea or severe clinical signs were observed. At necropsy, mild pulmonary congestion and multifocal petechial hemorrhages were observed in infected sheep, whereas no obvious gross lesions were detected in other organs. Histopathological examination revealed widening of the alveolar septa, thickening of the alveolar walls, vascular congestion, interstitial pulmonary edema, and mild mononuclear inflammatory cell infiltration in the lungs of infected animals (Figure 5). No significant histopathological lesions were observed in the lungs of the control sheep.

**Figure 6:**
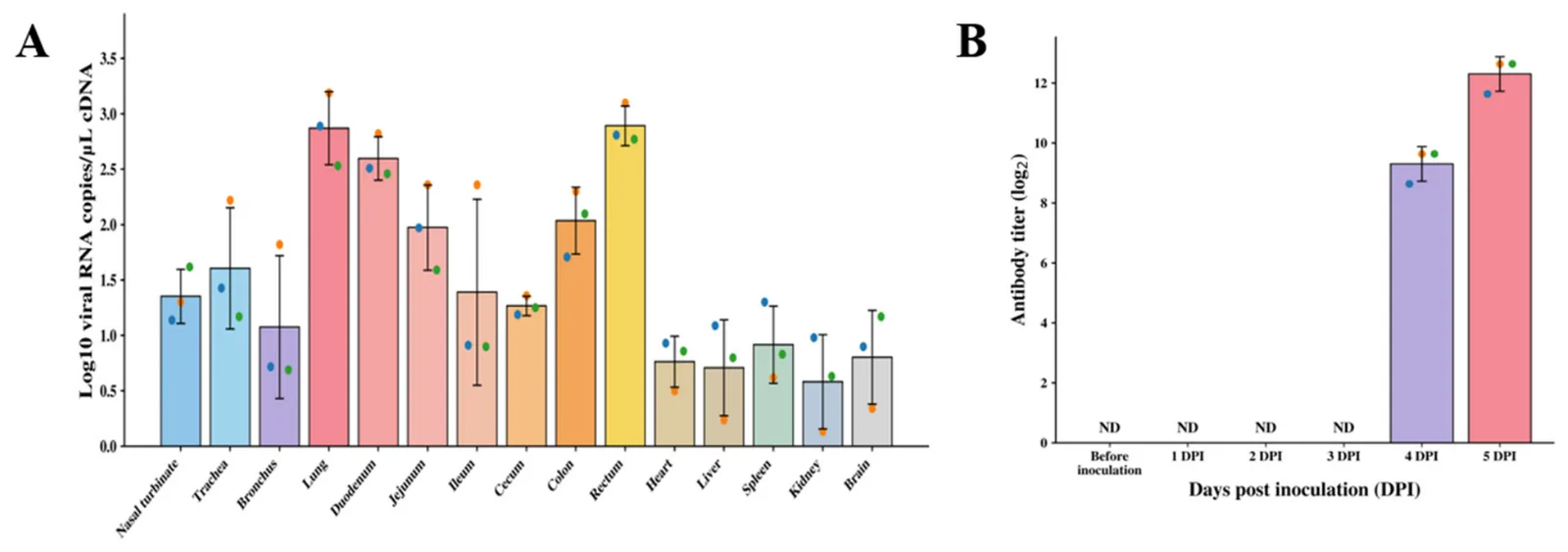
Tissue distribution of BCoV RNA and antibody responses in experimentally infected sheep. (**A**) Viral RNA loads in selected tissues collected at necropsy. Bars represent mean log_10_ viral RNA copies/μL cDNA ± SD from three infected sheep. (**B**) BCoV S2-reactive serum antibody titers measured by indirect ELISA after experimental infection. Bars represent mean log_2_ ELISA antibody titers ± SD from three infected sheep at 4 and 5 DPI. Colored dots represent individual data points. DPI, days post-infection.

BCoV RNA was detected in multiple tissues of infected sheep (Figure 6A). The highest mean viral RNA copy numbers were observed in the rectum and lung, with relatively high levels also detected in the duodenum, colon, and jejunum. Viral RNA was additionally detected in the trachea, nasal turbinate, bronchus, ileum, and cecum. Low levels of viral RNA were detected in the heart, liver, spleen, kidney, and brain. No BCoV RNA was detected in tissue samples collected from the control sheep.

Seroconversion was detected in all infected sheep, with BCoV-specific antibodies first becoming detectable at 4 DPI and increasing further by 5 DPI (Figure 6B). The control sheep remained seronegative throughout the experiment. In addition, BCoV was successfully re-isolated from lung tissues collected at necropsy, and the recovered virus showed the same sequence characteristics as the inoculated strain in the regions examined.

Viral RNA shedding was monitored by testing nasal swabs and fecal samples collected before inoculation and daily from 1 to 5 DPI. No BCoV RNA was detected before inoculation. Following inoculation, BCoV RNA was detected in nasal swabs and fecal samples at different time points during the observation period (Supplementary Table S5).

## 4. Discussion

Previous reports have provided evidence of BCoV-like particles, BCoV-reactive antibodies, or diagnostic detection of coronavirus in sheep, suggesting that sheep may be exposed to or infected by BCoV under natural conditions [14,15,16,21,22]. However, successful isolation of BCoV from sheep and genomic characterization of sheep-derived strains have not been reported. In the present study, two BCoV isolates were recovered from fecal samples of sheep during an outbreak involving respiratory and enteric signs in Qinghai Province, China, and nearly complete genome sequences were obtained. Experimental infection further demonstrated that the representative isolate could establish infection in sheep, as evidenced by viral RNA detection in tissues, seroconversion, and re-isolation of infectious virus from lung tissue. These findings provide the first isolation and genomic characterization of sheep-derived BCoV and provide evidence that the sheep-derived BCoV isolate was capable of infecting sheep. The availability of sheep-derived BCoV genomes also provides an important basis for investigating the genetic diversity, host range, and distribution of closely related BCoV lineages among different ruminant hosts. However, BCoV RNA was also detected in clinically healthy flockmates, indicating that subclinical infection may occur in sheep populations. Because the clinical samples in this study were collected during a naturally occurring outbreak, the numbers of clinically affected and healthy sheep were determined by sample availability rather than a prospectively designed sampling strategy, resulting in an unequal sample size between groups. Therefore, the association between BCoV detection and clinical status should be interpreted cautiously. Although BCoV infection was identified during the investigated outbreak, the contribution of additional host, environmental, or microbial factors to disease development cannot be excluded.

Experimental infection resulted mainly in mild respiratory manifestations, whereas no diarrhea was observed during the five-day observation period. Viral RNA was detected in both respiratory and intestinal tissues, with relatively high copy numbers observed in the rectum, lung, and duodenum. Similar findings have been reported in experimentally infected calves, in which respiratory signs predominated despite infection with enteric BCoV isolates [23]. In the natural outbreak investigated in this study, affected sheep showed both respiratory and enteric signs over a longer disease course. These observations suggest that sheep-derived BCoV can be detected in both respiratory and intestinal tissues following experimental infection, although clinical manifestations may vary depending on host factors, infection conditions, and observation periods. Low levels of viral RNA were additionally detected in several systemic tissues, including the heart, liver, spleen, kidney, and brain. However, the presence of viral RNA in these tissues should be interpreted cautiously because residual blood-associated viral RNA during tissue collection cannot be completely excluded. Because the present animal experiment involved a limited number of sheep and a short observation period, it was primarily designed to evaluate infectivity and host susceptibility rather than to fully define the pathogenic potential of sheep-derived BCoV. Therefore, the limited sample size restricted statistical evaluation, and the experimental findings should be interpreted accordingly.

The observed serological response further supported successful infection with the sheep-derived BCoV isolate. All experimentally infected sheep were negative for BCoV S2-reactive antibodies before inoculation, whereas antibody responses were detected after infection, indicating a BCoV-induced serological response. Previous studies on BCoV infection have shown that early antibody responses are mainly associated with IgM, with BCoV-specific IgM detected as early as 2 days post-infection in experimentally infected cattle and 4–9 days after disease onset during a natural outbreak [24,25]. A limitation of the present study is that early serum antibody responses were evaluated using rabbit anti-sheep IgG H&L as the secondary antibody. This assay design was selected primarily to determine whether the sheep-derived BCoV isolate could establish infection in sheep. However, this antibody was not pre-adsorbed to remove potential cross-reactivity with other sheep immunoglobulin classes; therefore, the contribution of IgM or other immunoglobulins to the detected signals cannot be excluded. Nevertheless, the observed responses demonstrate BCoV S2-reactive serum antibody responses following infection and support the establishment of infection in sheep. Further investigations into the immunological characteristics induced by this sheep-derived BCoV isolate will provide additional insights into host immune responses following infection.

One notable finding of this study is the extremely high genomic similarity between the sheep-derived isolates and the yak-derived strain Yak/SWUN/NF2/2022 previously identified in Qinghai Province. The complete coding regions of the sheep-derived isolates and Yak/SWUN/NF2/2022 shared 100% nucleotide identity, whereas differences were mainly observed at the genomic termini. Phylogenetic analyses further showed that BCoV strains from different host species generally did not form host-specific lineages, but instead tended to cluster with geographically related strains [13]. These findings suggest that closely related BCoV lineages may circulate among different ruminant hosts in the Qinghai–Tibet Plateau. However, the current data do not establish the direction or timing of potential interspecies transmission.

Although these substitutions should not be interpreted as sheep-specific molecular markers, the sheep-derived isolates and closely related Qinghai yak-derived strains shared lineage-associated substitutions in the spike protein, including M118R in the S1 subunit and M44T in the S2 subunit, with positions numbered relative to the corresponding subunits, as well as a shared substitution in the HE protein, P384S. The S protein mediates receptor recognition and membrane fusion, whereas HE is involved in receptor binding and receptor-destroying activity [4,5,6,7,8,9]. These amino acid mutations may represent lineage-associated genetic features; however, their effects on viral attachment, replication, host adaptation, or transmission remain unclear. Previous studies have suggested that genetic variation in coronavirus S and HE proteins may affect host range, tissue tropism, and interspecies transmission potential [4,9]. In this context, the shared substitutions observed in sheep- and yak-derived strains may reflect the evolutionary characteristics of this BCoV lineage circulating among local ruminant populations, although functional validation is required. The Qinghai–Tibet Plateau provides an ecological setting in which sheep, yak, wild ruminants, and rodents may share grazing habitats, water sources, or environmental exposure routes, creating opportunities for viral exchange among species. Previous reports have shown that BCoV or bovine-like coronaviruses can be detected in multiple domestic and wild ruminants [10,13,14,17,18,19], and BCoV has more recently been identified in rodents or shown to infect mice experimentally [11,12]. Thus, the unique or lineage-specific genetic features observed in the sheep- and yak-derived strains may reflect ongoing viral evolution in a multi-host high-altitude ecosystem. However, no direct functional evidence is currently available for these substitutions, and their biological significance remains unclear. Further studies are needed to determine whether these mutations influence receptor interaction, viral fitness, host adaptation, or transmission potential. Continued surveillance of BCoV in mixed-grazing systems will be important for clarifying its ecology, transmission dynamics, and evolutionary patterns in multi-host environments.

## 5. Conclusions

This study reports the first successful isolation and genomic characterization of BCoV from sheep and demonstrates that sheep-derived BCoV can establish infection in sheep. The extremely high genomic similarity between the sheep-derived isolates and a yak-derived strain previously identified in Qinghai Province, including 100% nucleotide identity across the complete coding regions, indicates a close genetic relationship between BCoV strains detected in different ruminant hosts. The shared lineage-associated substitutions identified in the sheep- and yak-derived strains may represent genetic features of this regional BCoV lineage, although their biological significance requires further investigation. These findings expand our understanding of the host range, genetic diversity, and distribution of BCoV lineages among ruminants and highlight the need for continued surveillance in mixed-grazing ecosystems.

## Data Availability

The genome sequences of the sheep-derived BCoV isolates generated in this study have been deposited in GenBank under accession numbers OR676674 and OR676675. Additional datasets supporting the findings of this study are available in the Zenodo repository (https://doi.org/10.5281/zenodo.22007829) [34]. Additional experimental data are provided in the Supplementary Materials.

## Supplementary Materials

The following supporting information can be downloaded at: https://www.mdpi.com/article/10.3390/ani16203276/s1, Table S1: Information on detection methods for common diarrhoeal pathogens; Table S2: Primers used for BCoV genome validation; Table S3: Metadata of BCoV reference sequences used in comparative analyses; Table S4: Individual Ct values of BCoV RNA detected in sheep samples; Table S5: Viral shedding detection in experimentally infected sheep; Table S6: Tissue distribution dataset; Table S7: ELISA dataset; Dataset S1: All 280 BCoV genome sequences (complete coding regions); Dataset S2: All 507 BCoV *S* gene sequences; Dataset S3: All 371 BCoV *HE* gene sequences; Dataset S4–S6: Representative sequence datasets used for phylogenetic analyses. Refs. [26,27,28,29,30,31,32,33] are cited in Supplementary Materials.

## References

[1] Saif L.J. (2010). Bovine respiratory coronavirus. Vet. Clin. N. Am. Food Anim. Pract..

[2] Zhu Q., Li B., Sun D. (2022). Advances in bovine coronavirus epidemiology. Viruses.

[3] Lai M.M., Cavanagh D. (1997). The molecular biology of coronaviruses. Adv. Virus Res..

[4] Li F. (2016). Structure, function, and evolution of coronavirus spike proteins. Annu. Rev. Virol..

[5] Peng G., Xu L., Lin Y.-L., Chen L., Pasquarella J.R., Holmes K.V., Li F. (2012). Crystal structure of bovine coronavirus spike protein lectin domain. J. Biol. Chem..

[6] Schultze B., Gross H.J., Brossmer R., Herrler G. (1991). The S protein of bovine coronavirus is a hemagglutinin recognizing 9-O-acetylated sialic acid as a receptor determinant. J. Virol..

[7] Kienzle T.E., Abraham S., Hogue B.G., Brian D.A. (1990). Structure and orientation of expressed bovine coronavirus hemagglutinin-esterase protein. J. Virol..

[8] Schultze B., Wahn K., Klenk H.-D., Herrler G. (1991). Isolated HE-protein from hemagglutinating encephalomyelitis virus and bovine coronavirus has receptor-destroying and receptor-binding activity. Virology.

[9] Bakkers M.J.G., Lang Y., Feitsma L.J., Hulswit R.J.G., de Poot S.A.H., van Vliet A.L.W., Margine I., de Groot-Mijnes J.D.F., van Kuppeveld F.J.M., Langereis M.A. (2017). Betacoronavirus adaptation to humans involved progressive loss of hemagglutinin-esterase lectin activity. Cell Host Microbe.

[10] Amer H.M. (2018). Bovine-like coronaviruses in domestic and wild ruminants. Anim. Health Res. Rev..

[11] Guo S., Yan T., Gao M., Zhou Y., Zhang Z., Liu Y., Zhang G., Zhu Z., Fan C. (2024). Study on the susceptibility of bovine coronavirus to BALB/c mice. Microb. Pathog..

[12] Xu L., Liu W., Bie M., Hu T., Yan D., Xiao Z., Holmes E.C., Shi W. (2023). Identification of bovine coronavirus in a daurian ground squirrel expands the host range of betacoronavirus 1. Virol. Sin..

[13] Alekseev K.P., Vlasova A.N., Jung K., Hasoksuz M., Zhang X., Halpin R., Wang S., Ghedin E., Spiro D., Saif L.J. (2008). Bovine-like coronaviruses isolated from four species of captive wild ruminants are homologous to bovine coronaviruses, based on complete genomic sequences. J. Virol..

[14] Burimuah V., Sylverken A., Owusu M., El-Duah P., Yeboah R., Lamptey J., Frimpong Y.O., Agbenyega O., Folitse R., Tasiame W. (2020). Sero-prevalence, cross-species infection and serological determinants of prevalence of bovine coronavirus in cattle, sheep and goats in ghana. Vet. Microbiol..

[15] Harp J.A., Myers L.L., Rich J.E., Gates N.L. (1981). Role of salmonella arizonae and other infective agents in enteric disease of lambs. Am. J. Vet. Res..

[16] Tzipori S., Smith M., Makin T., McCaughan C. (1978). Enteric coronavirus-like particles in sheep. Aust. Vet. J..

[17] Smith F.L., Heller M.C., Crossley B.M., Clothier K.A., Anderson M.L., Barnum S.S., Pusterla N., Rowe J.D. (2022). Diarrhea outbreak associated with coronavirus infection in adult dairy goats. J. Vet. Intern. Med..

[18] Chothe S.K., Byukusenge M., Sekhwal M.K., Li L., LaBella L.C., Jakka P., Palchak K., Barry R., Yon M., Nissly R.H. (2023). Complete genome sequence of a bovine coronavirus isolated from a goat in pennsylvania, USA. Microbiol. Resour. Announc..

[19] Mao L., Cai X., Li J., Li X., Li S., Li W., Lu H., Dong Y., Zhai J., Xu X. (2025). Discovery of a novel betacoronavirus 1, cpCoV, in goats in China: The new risk of cross-species transmission. PLoS Pathog..

[20] Bao C., Ren Y., Tang C., Su Y., Yue H., Chen X. (2026). Viral isolation and genomic characteristics of the first bovine parainfluenza virus type 3 isolated from water buffaloes (*Bubalus bubalis*) in China. Vet. J..

[21] Dahmani H., Ouchene N., Dahmani A., Ouchene-Khelifi N.A., Oumouna M. (2020). First report on cryptosporidium parvum, escherichia coli K99, rotavirus and coronavirus in neonatal lambs from north-center region, algeria. Comp. Immunol. Microbiol. Infect. Dis..

[22] Tråvén M., Carlsson U., Lundén A., Larsson B. (1999). Serum antibodies to bovine coronavirus in swedish sheep. Acta Vet. Scand..

[23] Cho H.-C., Kim Y., Cho Y.-I., Park J., Choi K.-S. (2024). Evaluation of bovine coronavirus in korean native calves challenged through different inoculation routes. Vet. Res..

[24] Tråvén M., Näslund K., Linde N., Linde B., Silván A., Fossum C., Hedlund K.O., Larsson B. (2001). Experimental reproduction of winter dysentery in lactating cows using BCV—Comparison with BCV infection in milk-fed calves. Vet. Microbiol..

[25] Näslund K., Tråvén M., Larsson B., Silván A., Linde N. (2000). Capture ELISA systems for the detection of bovine coronavirus-specific IgA and IgM antibodies in milk and serum. Vet. Microbiol..

[26] Yan N., Li R., Wang Y., Zhang B., Yue H., Tang C. (2020). High prevalence and genomic characteristics of G6P[1] Bovine Rotavirus A in yak in China. J. Gen. Virol..

[27] Gong X., Liu L., Zheng F., Chen Q., Li Z., Cao X., Yin H., Zhou J., Cai X. (2014). Molecular investigation of bovine viral diarrhea virus infection in yaks (*Bos gruniens*) from Qinghai, China. Virol. J..

[28] Jiménez-Clavero M.A., Escribano-Romero E., Mansilla C., Gómez N., Córdoba L., Roblas N., Ponz F., Ley V., Sáiz J.-C. (2005). Survey of bovine enterovirus in biological and environmental samples by a highly sensitive real-time reverse transcription-PCR. Appl. Environ. Microbiol..

[29] Abi K.M., Yu Z., Jing Z.Z., Tang C. (2021). Identification of a novel Aichivirus D in sheep. Infect. Genet. Evol..

[30] Salehi T.Z., Tadjbakhsh H., Atashparvar N., Nadalian M.G., Mahzounieh M.R. (2007). Detection and identification of Salmonella Typhimurium in bovine diarrhoeic fecal samples by immunomagnetic separation and multiplex PCR assay. Zoonoses Public Health.

[31] Yadegari Z., Nikbakht Brujeni G., Ghorbanpour R., Moosakhani F., Lotfollahzadeh S. (2019). Molecular characterization of enterotoxigenic Escherichia coli isolated from neonatal calves diarrhea. Vet. Res. Forum.

[32] Townsend K.M., Frost A.J., Lee C.W., Papadimitriou J.M., Dawkins H.J.S. (1998). Development of PCR assays for species- and type-specific identification of Pasteurella multocida isolates. J. Clin. Microbiol..

[33] Xiao L., Escalante L., Yang C., Sulaiman I., Escalante A.A., Montali R.J., Fayer R., Lal A.A. (1999). Phylogenetic analysis of Cryptosporidium parasites based on the small-subunit rRNA gene locus. Appl. Environ. Microbiol..

[34] Chen X. (2026). Data Supporting “First Isolation and Genomic Characterization of Bovine Coronavirus from Sheep”.

