# Supplementary material for: First Isolation, Genomic Characterization and Experimental Infection of Bovine Coronavirus from Sheep
Source: Animals (Basel). 2026 Oct 17;16(20):3276. doi: 10.3390/ani16203276 (PMC13700650; doi:10.3390/ani16203276)
Supplement: Supplementary file 1 [file animals-16-03276-s001.zip › Supplementary Tables S1-S5.pdf]

Supplementary Table S1 Information on detection methods for common diarrhoeal pathogens

The name of the pathogen	Primer sequences	Source of detection method
Bovine Rotavirus Group A	F: CCACCAGGTATGAATTGGAC<br>R: GAGTAATCACTCAGATGGCG	Yan N et al., 2020 [26]
Bovine Viral Diarrhea Virus	F: AGGCTAGCCATGCCCTTAGT<br>R: CTCCATGTGCCATGTACAGC	Gong X et al., 2014 [27]
Enterovirus	F: GGGGAGTAGTCCGACTCCGC<br>R: CAGAGCTACCGGGGTTGTGG	Jiménez-Clavero MA et al., 2005 [28]
Sheep Aichivirus D	F: AAGCCCTTGCTCTTTACACTTCTACCC<br>R: CACGGGTCAGCCCCTTCTTCAC	Abi KM et al., 2021 [29]
Salmonella	F: GTGAAATTATCGCCACGTTCGGGCAA<br>R: TCATCGCACCGTCAAAGGGAACC	Salehi TZ et al., 2007 [30]
Enterotoxigenic Escherichia coli	F: GGTATCCTTTAGCAGCAGTATTTC<br>R: GCATCAGCGGCAGTATCT	Yadegari Z et al., 2019 [31]
Pasteurellaceae	F: GCTGTAAACGAACTCGCCAC<br>R: ATCCGCTATTTACCCAGTGG	Townsend KM et al., 1998 [[32]
Cryptosporidium spp.	F: AACCTGGTTGATCCTGCCAGTAGTC<br>R: TGATCCTTCTGCAGGTTCACCTACG	Xiao L et al., 1999 [33]

Notes: The table contains primer information for 8 detection methods, including Rotavirus group A, Bovine viral diarrhea virus, Enterovirus, Sheep aichivirus D, *Salmonella*, *Enterotoxigenic Escherichia coli*, *Pasteurellaceae* and *Cryptosporidiumn spp*. The sources of the literature on the detection methods used for detecting pathogens are as follows:

Abi KM, Yu Z, Jing ZZ, et al. Identification of a novel Aichivirus D in sheep. Infect Genet Evol. 2021;91:104810.

Gong X, Liu L, Zheng F, et al. Molecular investigation of bovine viral diarrhea virus infection in yaks (Bos gruniens) from Qinghai, China. Virol J. 2014;11:29. Published 2014 Feb 14.

Jiménez-Clavero MA, Escribano-Romero E, Mansilla C, et al. Survey of bovine enterovirus in biological and environmental samples by a highly sensitive real-time reverse transcription-PCR. Appl Environ Microbiol. 2005;71(7):3536-3543.

Salehi TZ, Tadjbakhsh H, Atashparvar N, et al. Detection and identification of Salmonella Typhimurium in bovine diarrhoeic fecal samples by immunomagnetic separation and multiplex PCR assay. Zoonoses Public Health. 2007;54(6-7):231-236.

Townsend KM, Frost AJ, Lee CW, et al. Development of PCR assays for species- and type-specific identification of Pasteurella multocida isolates. J Clin Microbiol. 1998;36(4):1096-1100.

Xiao L, Escalante L, Yang C, et al. Phylogenetic analysis of Cryptosporidium parasites based on the small-subunit rRNA gene locus. Appl Environ Microbiol. 1999;65(4):1578-1583

Yadegari Z, Nikbakht Brujeni G, Ghorbanpour R, et al. Molecular characterization of enterotoxigenic Escherichia coli isolated from neonatal calves diarrhea. Vet Res Forum. 2019;10(1):73-78.

Yan N, Li R, Wang Y, et al. High prevalence and genomic characteristics of G6P[1] Bovine Rotavirus A in yak in China. J Gen Virol. 2020;101(7):701-711.

Supplementary Table S2 Primers used for BCoV genome validation.

Primer Name	Primer Sequence (5′→3′)	Amplified fragment size
1	F: TGCGTGCGTGCATCCCGCTT<br>R: CGCAACCAGCAATTATTGCG	3427 bp
2	F: GTATGTGCCTAAAGCAACTCGC<br>R: ACCTCTAGCAACCACACTCC	3403 bp
3	F: GGCTGGATCAAAATATCCGC<br>R: CTGAGAACAAGCAGCCTCC	3186 bp
4	F: CTGCTGCATATAGGGAGG<br>R: GGATTGTTCACAACCAGC	3958 bp
5	F: CGCAATGATTGCATGCTGC<br>R: ATAGCACTCCACCCTGACC	3405 bp
6	F: GACTGCACTCGTATTGTTCC<br>R: GCTGTCTTCGGTACAAGCC	3250 bp
7	F: GGACTATGCCTTTGAACAC<br>R: CTGGCATTGTTCCACCGAG	3002 bp
8	F: GCGTAAGGATTTTACGCCTG<br>R: GGAGGCAACACCTTGATACC	3335 bp
9	F: GGGATCTCATTTGTGTGC<br>R: CTGGATTGCTTACCAGGAG	2947 bp
10	F: CTGGCTTGGACACCGCATTG<br>R: GGCACTTGTCGGCAATTAC	1600 bp

Supplementary Table S3. Metadata of BCoV reference sequences used in comparative analyses

Complete coding regions (280 sequences)

Accession number	Strain name	Country/Region
OR676674.1_	Ovine/SWUN/FA/2023	China
OR676675.1_	Bovine/SWUN/28-3/2023	China
OR603986.1_	Yak/SWUN/NF2/2022	China
OR603987.1_	Yak/SWUN/NF5/2022	China
OR596700.1_	BCoV/SWUN/XHD2/2022	China
OQ718861.1_	BCoV/SWUN/XHD5/2022	China
OR612023.1_	BCoV/SWUN/HXD2/2022	China
OR612024.1_	BCoV/SWUN/HXD3/2022	China
OR612025.1_	BCoV/SWUN/HXD4/2022	China
OR612026.1_	BCoV/SWUN/HXD6/2022	China
OR612027.1_	BCoV/SWUN/HXD7/2022	China
OR621177.1_	BCoV/SWUN/XHD3/2022	China
OR621178.1_	BCoV/SWUN/XHD4/2022	China
OR621176.1_	BCoV/SWUN/XHD1/2022	China
OR603989.1_	BCoV/SWUN/HXD5/2022	China
OR612022.1_	BCoV/SWUN/HXD1/2022	USA
OR612020.1_	BCoV/SWUN/XHD6/2022	China
OR612021.1_	BCoV/SWUN/XHD5/2022	China
OR621174.1_	BCoV/SWUN/XHD7/2022	China
OR612019.1_	BCoV/SWUN/HXD10/2022	China
OR621175.1_	BCoV/SWUN/HXD8/2022	China
OP004056.2_	CH-4-22-01	USA
OR077308.1_	RG23F3	China
OR077316.1_	JSYZ2209	China
OR077315.1_	HBSJZ2202	China
OR077313.1_	GZBJ2207	China
OR750853.1_	BCoV-XJ-SHZ-37	China
OR077306.1_	HMsz2207	China
OR077307.1_	ZJ2303G	China
OR088593.1_	BCoV/YN1LC/2023	China
OR077309.1_	AHFY2302G	China
OR454204.1_	BCoV/YNZT/2023	China
OR077310.1_	YC2304G	China
OR077311.1_	XJCJ2301G	China
OR077312.1_	HBLF2302	China
OR077314.1_	HBSJZ2112	China
OR077317.1_	ZJNB2106	China
OR947456.1_	BCoV/NX-1/CHN/2022	China
MZ100070.1_	Tapir_coronavirus_1044512-1	USA

MG518518.1_	Water_deer_W17-18	Korea
FJ425187.1_	White-tailed_deer_US/OH-WD470/1994	USA
EF424623.1_	Giraffe_coronavirus_US/OH3/2003	USA
FJ425189.1_	Sambar_deer_coronavirus_US/OH-WD388/1994	USA
EF424621.1_	Sable_antelope_coronavirus_US/OH1/2003	USA
FJ425184.1_	Waterbuck_coronavirus_US/OH-WD358-TC/1994	USA
FJ425188.1_	Sambar_deer_coronavirus_US/OH-WD388-TC/1994	USA
ON014593.1_	Bovine-like_Dama_M0032	UK
FJ425190.1_	Sambar_deer_US/OH-WD388-TC/1994	USA
EF424622.1_	Giraffe_US/OH3-TC/2006	USA
EF424624.1_	Calf-giraffe_US/OH3/2006	USA
FJ425186.1_	Waterbuck_US/OH-WD358/1994	USA
FJ425185.1_	Waterbuck_US/OH-WD358-GnC/1994	USA
DQ915164.2_	Alpaca	USA
OP037427.1_	VDC_2018_12_E	USA_Nebraska
AF391541.1_	BCoV-ENT	unknown
AF220295.1_	Quebec	unknown
AF391542.1_	BCoV-LUN	unknown
U00735.2_	Mebus	unknown
EF424615.1_	E-AH65	USA_Ohio
EF424616.1_	E-AH65-TC	USA_Ohio
EF424617.1_	R-AH65	USA_Ohio
EF424618.1_	R-AH65-TC	USA_Ohio
EF424619.1_	E-AH187	USA_Ohio
EF424620.1_	R-AH187	USA_Ohio
EF424621.1_	US_OH1_2003	USA_Ohio
EF424622.1_	US_OH3-TC_2006	USA_Ohio
EF424623.1_	US_OH3_2003	USA_Ohio
EF424624.1_	Calf-US_OH3_2006	USA_Ohio
AB354579.1_	genomic_RNA_strain_Kakegawa	Japan
FJ415324.1_	Human_enteric_coronavirus_4408	Germany
FJ425184.1_	US_OH-WD358-TC_1994	USA_Ohio
FJ425185.1_	US_OH-WD358-GnC_1994	USA_Ohio
LC494130.1_	IWT-2_RNA	Japan
LC494131.1_	IWT-3_RNA	Japan
LC494132.1_	IWT-4_RNA	Japan
LC494133.1_	IWT-5_RNA	Japan
FJ425186.1_	US_OH-WD358_1994	USA_Ohio
FJ425187.1_	US_OH-WD470_1994	USA_Ohio
FJ425188.1_	US_OH-WD388-TC_1994	USA_Ohio
FJ425189.1_	US_OH-WD388_1994	USA_Ohio

FJ425190.1_	US_OH-WD388-TC_1994	USA_Ohio
FJ938063.1_	E-DB2-TC	USA_Ohio
FJ938064.1_	E-AH187-TC	USA_Ohio
FJ938065.1_	AH187	USA_Ohio
FJ938066.1_	bovine_US_OH-440-TC_1996	USA_Ohio
FJ938067.1_	Human_enteric_coronavirus_strain_4408	unknown
KX432213.1_	BJ232	China
MG757139.1_	ICSA16-EN	France
LC494138.1_	IWT-12_RNA	Japan
DQ811784.2_	DB2	unknown
KU886219.1_	BCV-AKS-01	China_Xinjiang
LQ289146.1_	Sequence_25_from_Patent_WO2016034610	unknown
LC494127.1_	IWT-9_RNA	Japan
LC494128.1_	IWT-10_RNA	Japan
KX982264.1_	BCoV_2014_13	France
MG757138.1_	ICSA21-LBA	France
MG757141.1_	ICSA-pool-EN	France
MG757140.1_	ICSA16-LBA	France
MG757142.1_	ICSA-pool-LBA	France
LC494140.1_	IWT-14_RNA	Japan
LC494141.1_	IWT-15_RNA	Japan
LC494142.1_	IWT-18_RNA	Japan
LC494143.1_	IWT-19_RNA	Japan
LC494150.1_	IWT-24_RNA	Japan
LC494144.1_	IWT-20_RNA	Japan
LC494156.1_	SHG-3_RNA	Japan
LC494157.1_	SHG-4_RNA	Japan
LC494158.1_	SHG-5_RNA	Japan
LC494145.1_	IWT-21_RNA	Japan
LC494161.1_	TCG-5_RNA	Japan
LC494146.1_	IWT-16_RNA	Japan
LC494160.1_	TCG-4_RNA	Japan
LC494170.1_	TCG-15_RNA	Japan
LC494171.1_	TCG-16_RNA	Japan
LC494172.1_	TCG-6_RNA	Japan
LC494173.1_	TCG-7_RNA	Japan
MH043954.1_	4-17-08	USA_Pennsylvania
MH043952.1_	4-17-03	USA_Pennsylvania
MG518518.1_	W17-18	South_Korea_Chungnam
MH043953.1_	4-17-25	USA_Pennsylvania
MH043955.1_	7-16-23	USA_Pennsylvania
MH810163.1_	Yak_coronavirus_strain_YAK_HY24_CH_2017	China
LC494179.1_	TCG-20_RNA	Japan

LC494180.1_	TCG-21_RNA	Japan
LC494181.1_	TCG-22_RNA	Japan
LC494182.1_	TCG-23_RNA	Japan
LC494183.1_	TCG-24_RNA	Japan
LC494184.1_	TCG-25_RNA	Japan
LC494185.1_	TCG-26_RNA	Japan
LC494186.1_	TCG-27_RNA	Japan
LC494187.1_	TCG-28_RNA	Japan
LC494188.1_	TCG-29_RNA	Japan
LC494189.1_	TCG-30_RNA	Japan
LC494190.1_	TCG-31_RNA	Japan
LC494191.1_	TCG-32_RNA	Japan
LC494192.1_	TCG-33_RNA	Japan
LC494126.1_	GIF-1_RNA	Japan
LC494129.1_	IWT-1_RNA	Japan
LC494134.1_	IWT-6_RNA	Japan
LC494135.1_	IWT-7_RNA	Japan
LC494167.1_	TCG-12_RNA	Japan
LC494169.1_	TCG-14_RNA	Japan
LC494136.1_	IWT-8_RNA	Japan
LC494137.1_	IWT-11_RNA	Japan
LC494139.1_	IWT-13_RNA	Japan
LC494147.1_	IWT-17_RNA	Japan
LC494159.1_	SHG-6_RNA	Japan
LC494162.1_	TCG-3_RNA	Japan
LC494163.1_	TCG-2_RNA	Japan
LC494164.1_	TCG-1_RNA	Japan
LC494148.1_	IWT-22_RNA	Japan
LC494149.1_	IWT-23_RNA	Japan
LC494151.1_	IWT-25_RNA	Japan
LC494152.1_	IWT-26_RNA	Japan
LC494153.1_	IWT-27_RNA	Japan
OP037418.1_	VDC_2018_03_R	USA_Nebraska
LC494154.1_	SHG-1_RNA	Japan
LC494155.1_	SHG-2_RNA	Japan
LC494165.1_	TCG-10_RNA	Japan
LC494166.1_	TCG-11_RNA	Japan
LC494168.1_	TCG-13_RNA	Japan
LC494174.1_	TCG-8_RNA	Japan
LC494175.1_	TCG-18_RNA	Japan
LC494176.1_	TCG-17_RNA	Japan
LC494177.1_	TCG-9_RNA	Japan
LC494178.1_	TCG-19_RNA	Japan

LC642814.1_	BoCoV_GF2020_RNA	Japan
ON146444.1_	MARC_BRCV_2014	USA_Nebraska
ON093194.1_	BCoV2_2021_CHN	China
ON792941.1_	ABGEB0-52	Ireland
ON792942.1_	ABGEB0-54	Ireland
ON792943.1_	ABGEB0-56	Ireland
ON792944.1_	ABGEB0-59	Ireland
ON792945.1_	ABGEB0-62	Ireland
ON792946.1_	ABGEB0-66	Ireland
ON792947.1_	ABGEB0-70	Ireland
ON792948.1_	ABGEB0-72	Ireland
ON792949.1_	ABGEB0-74	Ireland
ON792950.1_	ABGEB0-78	Ireland
ON792951.1_	ABGEB0-79	Ireland
ON792963.1_	ABGEDB-205	Ireland
MO401628.1_	Sequence_25_from_patent_US_10434168	unknown
MN982198.1_	BCOV-China_SWUN_A1_2018	China
MW711287.1_	SWUN_NMG-D10_2020	China
ON792964.1_	ABGEDB-260	Ireland
OP037392.1_	MARC_2020_02_R	USA_Nebraska
ON792952.1_	ABGEB0-81	Ireland
ON792953.1_	ABGEB0-88	Ireland
ON792954.1_	ABGEB0-110	Ireland
ON792955.1_	ABGEB0-116	Ireland
ON792956.1_	ABGEB0-122	Ireland
ON792957.1_	ABGEB0-128	Ireland
ON792958.1_	ABGEBB-78	Ireland
ON792959.1_	ABGEBB-108	Ireland
ON792960.1_	ABGEBB-110	Ireland
ON792961.1_	ABGEBB-115	Ireland
ON792962.1_	ABGEBB-129	Ireland
OP037365.1_	MARC_2013_01_R	USA_Nebraska
OP037366.1_	MARC_2013_02_R	USA_Nebraska
OP037367.1_	MARC_2014_01_R	USA_Nebraska
OP037368.1_	MARC_2014_02_R	USA_Nebraska
OP037369.1_	MARC_2014_03_R	USA_Nebraska
OP037370.1_	MARC_2014_04_R	USA_Nebraska
OP037371.1_	MARC_2015_01_R	USA_Nebraska
OP037372.1_	MARC_2015_02_R	USA_Nebraska
OP037373.1_	MARC_2016_01_R	USA_Nebraska
OP037374.1_	MARC_2016_02_R	USA_Nebraska
OP037375.1_	MARC_2016_03_R	USA_Nebraska
OP037376.1_	MARC_2016_04_R	USA_Nebraska

OP037406.1_	SDSU_2021_03_R	USA_Texas
OP037407.1_	SDSU_2021_04_R	USA_Texas
OP037408.1_	SDSU_2021_05_R	USA_California
OP037409.1_	SDSU_2021_06_R	USA_North_Dakota
OP037377.1_	MARC_2016_05_R	USA_Nebraska
OP037394.1_	MARC_2021_01_R	USA_Nebraska
OP037395.1_	MARC_2021_02_R	USA_Nebraska
OP037396.1_	MARC_2021_03_R	USA_Nebraska
OP037403.1_	SDSU_2020_07_R	USA_Minnesota
OP037397.1_	SDSU_2020_01_R	USA_California
OP037378.1_	MARC_2016_06_R	USA_Nebraska
OP037379.1_	MARC_2016_07_R	USA_Nebraska
OP037380.1_	MARC_2017_01_R	USA_Nebraska
OP037381.1_	MARC_2017_02_E	USA_Nebraska
OP037382.1_	MARC_2017_03_E	USA_Nebraska
OP037399.1_	SDSU_2020_03_R	USA_Texas
OP037401.1_	SDSU_2020_05_R	USA_Minnesota
OP037383.1_	MARC_2017_04_E	USA_Nebraska
OP037384.1_	MARC_2017_05_R	USA_Nebraska
OP037385.1_	MARC_2017_06_R	USA_Nebraska
OP037386.1_	MARC_2018_01_R	USA_Nebraska
OP037387.1_	MARC_2018_02_R	USA_Nebraska
OP037388.1_	MARC_2018_05_E	USA_Nebraska
OP037389.1_	MARC_2019_01_R	USA_Nebraska
OP037390.1_	MARC_2019_02_R	USA_Nebraska
OP037414.1_	VDC_2017_01_E	USA_Nebraska
OP037415.1_	VDC_2017_02_EN	USA_Nebraska
OP037416.1_	VDC_2018_01_R	USA_Nebraska
OP037417.1_	VDC_2018_02_R	USA_Wyoming
OP037419.1_	VDC_2018_04_R	USA_Nebraska
OP037420.1_	VDC_2018_05_R	USA_Nebraska
OP037421.1_	VDC_2018_06_E	USA_Nebraska
OP037422.1_	VDC_2018_07_E	USA_Nebraska
OP037423.1_	VDC_2018_08_E	USA_Pennsylvania
OP037424.1_	VDC_2018_09_E	USA_Nebraska
OP037425.1_	VDC_2018_10_E	USA_Nebraska
OP037426.1_	VDC_2018_11_E	USA_Nebraska
OP037428.1_	VDC_2018_13_E	USA_Nebraska
OP037429.1_	VDC_2019_01_E	USA_Nebraska
OP037430.1_	VDC_2019_01_R	USA_Nebraska
OP037431.1_	VDC_2019_02_E	USA_Nebraska
OP037432.1_	VDC_2019_03_E	USA_Nebraska
OP037433.1_	VDC_2019_04_E	USA_Nebraska

OP037434.1_	VDC_2019_05_E	USA_Nebraska
OP037435.1_	VDC_2019_06_E	USA_Nebraska
OP037436.1_	VDC_2022_01_E	USA_Kansas
ON142317.1_	BCoV4_2021_CHN	China
OP037437.1_	VDC_2022_02_E	USA_South_Dakota
OP037438.1_	VDC_2022_03_E	USA_South_Dakota
OP037439.1_	VDC_2022_04_E	USA_Nebraska
OP037441.1_	VDC_2022_06_E	USA_Nebraska
OP037442.1_	VDC_2022_07_E	USA_Oregon
ON142315.1_	BCoV1_2021_CHN	China
ON142316.1_	BCoV3_2021_CHN	China
ON142318.1_	UNVERIFIED_BCoV5_2021_CHN	China
ON142319.1_	BCoV6_2021_CHN	China
ON142320.1_	BCoV7_2021_CHN	China
OP296992.1_	p95	Taiwan
OP037391.1_	MARC_2020_01_R	USA_Nebraska
OP037393.1_	MARC_2020_03_R	USA_Nebraska
OP037398.1_	SDSU_2020_02_R	USA_California
OP037404.1_	SDSU_2021_01_R	USA_Colorado
OP037405.1_	SDSU_2021_02_R	USA_Nebraska
OP037410.1_	SDSU_2022_01_R	USA_Nebraska
OP037440.1_	VDC_2022_05_E	USA_Nebraska
OP037411.1_	SDSU_2022_02_R	USA_Nebraska
OP037412.1_	SDSU_2022_03_R	USA_Nebraska
OP037413.1_	SDSU_2022_04_R	USA_Nebraska
MN982199.1_	BCOV-China_SWUN_A10_2018	China
OP037400.1_	SDSU_2020_04_R	USA_Wisconsin
OP037402.1_	SDSU_2020_06_R	USA_Nebraska

S gene sequences (507 sequences)

Accession number	Strain name	Country/Region
OR676674.1_	Ovine/SWUN/FA/2023	China
OR676675.1_	Ovine/SWUN/28-3/2023	China
OR603986.1_	Yak/SWUN/NF2/2022	China
OR603987.1_	Yak/SWUN/NF5/2022	China
OR077308.1_	RG23F3	China
OR077306.1_	HMsz2207	China
OR077307.1_	ZJ2303G	China
OR077310.1_	YC2304G	China
OR077309.1_	AHFY2302G	China
OR077311.1_	XJCJ2301G	China
OR612023.1_	BCoV/SWUN/HXD2/2022	China
OR612024.1_	BCoV/SWUN/HXD3/2022	China
OR612025.1_	BCoV/SWUN/HXD4/2022	China
OR612026.1_	BCoV/SWUN/HXD6/2022	China
OR612027.1_	BCoV/SWUN/HXD7/2022	China
OR621177.1_	BCoV/SWUN/XHD3/2022	China
OR621178.1_	BCoV/SWUN/XHD4/2022	China
OR603989.1_	BCoV/SWUN/HXD5/2022	China
OR621176.1_	BCoV/SWUN/XHD1/2022	China
OR603988.1_	BCoV/SWUN/HXD9/2022	China
OR612022.1_	BCoV/SWUN/HXD1/2022	USA
OR612020.1_	BCoV/SWUN/XHD6/2022	China
OR621174.1_	BCoV/SWUN/XHD7/2022	China
OR612019.1_	BCoV/SWUN/HXD10/2022	China
OR596700.1_	BCoV/SWUN/XHD2/2022	China
OR612021.1_	BCoV/SWUN/XHD5/2022	China
OR621175.1_	BCoV/SWUN/HXD8/2022	China
OR077312.1_	HBLF2302	China
OR077314.1_	HBSJZ2112	China
OR077317.1_	ZJNB2106	China
OR077316.1_	JSYZ2209	China
OR077315.1_	HBSJZ2202	China
OR077313.1_	GZBJ2207	China
OR750853.1_	BCoV-XJ-SHZ-37	China
OP004056.2_	CH-4-22-01	USA
OR753439.1_	BCo/YNLP/2023	China
OR088593.1_	BCoV/YN1LC/2023	China
OP037377.1_	MARC/2016/05/R	USA
OP037383.1_	MARC/2017/04/E	USA
OR454204.1_	BCoV/YNZT/2023	China

OR947456.1_	BCoV/NX-1/CHN/2022	China
DQ915164.2_	Alpaca(2)	USA
FJ425187.1_	White-tailed_deer_US/OH-WD470/1994	USA
EF424623.1_	Giraffe_coronavirus_US/OH3/2003	USA
FJ425189.1_	Sambar_deer_coronavirus_US/OH-WD388/1994	USA
EF424621.1_	Sable_antelope_coronavirus_US/OH1/2003	USA
FJ425184.1_	Waterbuck_coronavirus_US/OH-WD358-TC/1994	USA
FJ425188.1_	Sambar_deer_coronavirus_US/OH-WD388-TC/1994	USA
ON014593.1_	Bovine-like_Dama_M0032	UK
FJ425190.1_	Sambar_deer_US/OH-WD388-TC/1994	USA
FJ425186.1_	Waterbuck_US/OH-WD358/1994	USA
FJ425185.1_	Waterbuck_US/OH-WD358-GnC/1994	USA
MZ100070.1_	Tapir_coronavirus_1044512-1	USA
MW711296.1_	SWUN_NMG-D10_2020	China
AY935637.1_	KWD1	South_Korea
AY935638.1_	KWD2	South_Korea
AY935639.1_	KWD3	South_Korea
AY935640.1_	KWD4	South_Korea
AY935641.1_	KWD5	South_Korea
AY935642.1_	KWD6	South_Korea
AY935643.1_	KWD7	South_Korea
AY935644.1_	KWD8	South_Korea
AY935645.1_	KWD9	South_Korea
AY935646.1_	KWD10	South_Korea
DQ389632.1_	KCD1	South_Korea
DQ389633.1_	KCD2	South_Korea
DQ389634.1_	KCD3	South_Korea
DQ389635.1_	KCD4	South_Korea
DQ389636.1_	KCD5	South_Korea
DQ389637.1_	KCD6	South_Korea
DQ389638.1_	KCD7	South_Korea
DQ389639.1_	KCD8	South_Korea
DQ389640.1_	KCD9	South_Korea
DQ389641.1_	KCD10	South_Korea
DQ389652.1_	KWD11	South_Korea
DQ389653.1_	KWD12	South_Korea
DQ389654.1_	KWD13	South_Korea
DQ389655.1_	KWD14	South_Korea
DQ389656.1_	KWD15	South_Korea
DQ389657.1_	KWD16	South_Korea
DQ389658.1_	KWD17	South_Korea
DQ389659.1_	KWD18	South_Korea
DQ389660.1_	KWD19	South_Korea

EF193075.1_	V270	Germany
EU401986.1_	502	South_Korea
EU401987.1_	A3	South_Korea
EU401988.1_	SUN5	South_Korea
EU401989.1_	BC94	South_Korea
EU686689.1_	501	South_Korea
EU814647.1_	438_06-TN	Italy
EU814648.1_	438_06-TN-50	Italy
HE616738.1_	VB_7_09_MAYABEQUE	unknown
HE616739.1_	VB_16_10_CIENFUEGOS	unknown
HE616740.1_	VB_24_11_CIEGO_DE_AVILA	unknown
HE616741.1_	VB_68_11_CAMAGUEY	unknown
HM573326.1_	Wisent_10_01	South_Korea
HM573327.1_	Himalayan_Tahr1_10_01	South_Korea
HM573328.1_	Himalayan_Tahr2_10_01	South_Korea
HM573329.1_	Sitatunga_10_01	South_Korea
HM573330.1_	Nyala_10_01	South_Korea
KF169908.1_	SWE_C_92	Sweden
KF169909.1_	SWE_02-1	Sweden
KF169910.1_	SWE_02-2	Sweden
KF169911.1_	SWE_02-3	Sweden
KF169912.1_	SWE_02-4	Sweden
KF169913.1_	DEN_03-1	Denmark
KF169914.1_	DEN_03-2	Denmark
KF169915.1_	DEN_03-3	Denmark
KF169916.1_	DEN_05-1	Denmark
KF169917.1_	DEN_05-2	Denmark
KF169918.1_	DEN_05-3	Denmark
KF169919.1_	DEN_05-4	Denmark
KF169920.1_	SWE_N_05-1	Sweden
KF169921.1_	SWE_N_05-2	Sweden
KF169922.1_	SWE_AC_06-1	Sweden
KF169923.1_	SWE_M_06-3*	Sweden
KF169924.1_	SWE_M_06-4*	Sweden
KF169925.1_	SWE_Z_07-1	Sweden
KF169926.1_	SWE_C_07-2	Sweden
KF169927.1_	SWE_I_07-3	Sweden
KF169928.1_	SWE_I_07-4	Sweden
KF169929.1_	SWE_I_07-5	Sweden
KF169930.1_	SWE_C_07-6	Sweden
KF169931.1_	SWE_AC_08-1	Sweden
KF169932.1_	SWE_C_08-2	Sweden
KF169933.1_	SWE_I_08-3	Sweden

KF169934.1_	SWE_P_09-1	Sweden
KF169935.1_	SWE_C_09-2	Sweden
KF169936.1_	SWE_U_09-3*	Sweden
KF169937.1_	SWE_M_10-1	Sweden
KF169938.1_	SWE_M_10-2	Sweden
KF169939.1_	SWE_Y_10-3	Sweden
KF169940.1_	SWE_P_10-4	Sweden
KF272919.1_	RVLC7	Ireland
KM985631.1_	HLJ-14_CHN	China
KT318111.1_	BCoV_FRA_EPI_Caen_2005_01	France
KT318112.1_	BCoV_FRA_EPI_Caen_2005_02	France
KT318113.1_	BCoV_FRA_EPI_Caen_2007_03	France
KT318114.1_	BCoV_FRA_EPI_Caen_2008_04	France
KT318115.1_	BCoV_FRA_EPI_Caen_2003_05	France
KT318116.1_	BCoV_FRA_EPI_Caen_2010_06	France
KT318117.1_	BCoV_FRA_EPI_Caen_2012_07	France
KT318118.1_	BCoV_FRA_EPI_Caen_2013_08	France
KT318119.1_	BCoV_FRA_EPI_Caen_2013_09	France
KT318120.1_	BCoV_FRA_EPI_Caen_2013_10	France
KT318121.1_	BCoV_FRA_EPI_Caen_2013_11	France
KT318122.1_	BCoV_FRA_EPI_Caen_2014_12	France
KT318123.1_	BCoV_FRA_EPI_Caen_2014_13	France
KT318124.1_	BCoV_FRA_EPI_Caen_2004_14	France
MG757143.1_	strain_ICSA4-EN	France
MG757144.1_	strain_ICSA17-LBA	France
MH197037.1_	PL84	Viet_Nam
MH197038.1_	PL83	Viet_Nam
MH197039.1_	ND65	Viet_Nam
MH203064.1_	DT97	Viet_Nam
MH203065.1_	BL104	Viet_Nam
MH203066.1_	MC199	Viet_Nam
MH203067.1_	VP200	Viet_Nam
MH741424.1_	Yak_coronavirus_isolate_HY24	China
MK046003.1_	NA226	Viet_Nam
MK046004.1_	HT293	Viet_Nam
MK046005.1_	HCM304	Viet_Nam
MK046006.1_	HCM305	Viet_Nam
MK046007.1_	HCM306	Viet_Nam
MK046008.1_	HCM307	Viet_Nam
MK046009.1_	HT315	Viet_Nam
MK046010.1_	HT316	Viet_Nam
MK046011.1_	HT317	Viet_Nam
MK095174.1_	BCOV-China_SWUN_SC1_2017	China

MK095175.1_	BCOV-China_SWUN_SC2_2017	China
MK095176.1_	BCOV-China_SWUN_SC3_2017	China
MK095177.1_	BCOV-China_SWUN_SX1_2018	China
MK095178.1_	BCOV-China_SWUN_SX2_2018	China
MK095179.1_	BCOV-China_SWUN_LN1_2018	China
MK095180.1_	BCOV-China_SWUN_LN2_2018	China
MK095181.1_	BCOV-China_SWUN_LN3_2018	China
MK095182.1_	BCOV-China_SWUN_LN4_2018	China
MK095183.1_	BCOV-China_SWUN_LN5_2018	China
MK095184.1_	BCOV-China_SWUN_HN1_2018	China
MK095185.1_	BCOV-China_SWUN_HN2_2018	China
MK095186.1_	BCOV-China_SWUN_HN3_2018	China
MK903506.1_	BCoV_CH_HB-BD_2019	unknown
MN982166.1_	BCOV-China_SWUN_A2_2018	China
MN982167.1_	BCOV-China_SWUN_A3_2018	China
MN982168.1_	BCOV-China_SWUN_A4_2018	China
MN982169.1_	BCOV-China_SWUN_A5_2018	China
MN982170.1_	BCOV-China_SWUN_A6_2018	China
MN982171.1_	BCOV-China_SWUN_A7_2018	China
MN982172.1_	BCOV-China_SWUN_A8_2018	China
MN982173.1_	BCOV-China_SWUN_A9_2018	China
MN982174.1_	BCOV-China_SWUN_B1_2018	China
MN982175.1_	BCOV-China_SWUN_B2_2018	China
MN982176.1_	BCOV-China_SWUN_B3_2018	China
MN982177.1_	BCOV-China_SWUN_B4_2018	China
MN982178.1_	BCOV-China_SWUN_B5_2018	China
MN982179.1_	BCOV-China_SWUN_B6_2018	China
MN982180.1_	BCOV-China_SWUN_B9_2018	China
MN982181.1_	BCOV-China_SWUN_B10_2018	China
MT975570.1_	BCoV-GX-FS191104	China
MT975571.1_	BCoV-GX-HC181120	China
MT975572.1_	BCoV-GX-NN190313	China
MW521175.1_	HLJ_MDJ-1_2020	China
MW521176.1_	HLJ_QQHR-2_2020	China
MW521177.1_	HLJ_QQHR-3_2020	China
MW521178.1_	HLJ_QQHR-4_2020	China
MW521179.1_	HLJ_QQHR-5_2020	China
MW521180.1_	HLJ_QQHR-6_2020	China
MW521181.1_	HLJ_QQHR-7_2020	China
MW521182.1_	HLJ_QQHR-8_2020	China
MW521183.1_	HLJ_DQ-9_2020	China
MW521184.1_	HLJ_HH-10_2020	China
MW521185.1_	HLJ_HH-11_2020	China

MW521186.1_	HLJ_HH-12_2020	China
MW521187.1_	HLJ_HH-13_2020	China
MW521188.1_	HLJ_HH-14_2020	China
MW521189.1_	HLJ_HH-15_2020	China
MW521190.1_	HLJ_HH-16_2020	China
MW521191.1_	HLJ_HH-17_2020	China
MW521192.1_	HLJ_HH-18_2020	China
MW521193.1_	HLJ_HH-19_2020	China
MW521194.1_	HLJ_HH-20_2020	China
MW711289.1_	SWUN_HN-2-B12_2019	China
MW711290.1_	SWUN_HN-KF3_2019	China
MW711291.1_	SWUN_NX-YC10_2019	China
MW711292.1_	SWUN_NX-YCP7_2019	China
MW711293.1_	SWUN_NX-YCP9_2019	China
MW711294.1_	SWUN_XC-7_2020	China
MW711295.1_	SWUN_NMG-D7_2020	China
MW711297.1_	SWUN_NMG-4_2020	China
MW711298.1_	SWUN_NMG-15_2020	China
MW711299.1_	SWUN_NMG-17_2020	China
MW711300.1_	SWUN_NMG-7_2020	China
MW711301.1_	SWUN_NMG-8_2020	China
MW711302.1_	SWUN_NMG-9_2020	China
MW711303.1_	SWUN_NMG-10_2020	China
MW711304.1_	SWUN_NMG-13_2020	China
MZ711344.1_	BCoV_HLJ1_CHN_2020	China
MZ711345.1_	BCoV_HLJ2_CHN_2020	China
MZ711346.1_	BCoV_HLJ9_CHN_2020	China
MZ711347.1_	BCoV_HLJ12_CHN_2020	China
MZ711348.1_	BCoV_JS3_CHN_2020	China
MZ711349.1_	BCoV_JS13_CHN_2020	China
MZ711350.1_	BCoV_JS33_CHN_2020	China
MZ711351.1_	BCoV_JS46_CHN_2020	China
MZ711352.1_	BCoV_NM14_CHN_2020	China
MZ711353.1_	BCoV_NM24_CHN_2020	China
MZ711354.1_	BCoV_NM30_CHN_2020	China
MZ711355.1_	BCoV_NM31_CHN_2020	China
MZ711356.1_	BCoV_NM45_CHN_2020	China
MZ711357.1_	BCoV_NM46_CHN_2020	China
MZ711358.1_	BCoV_ShX240_CHN_2021	China
MZ711359.1_	BCoV_ShX310_CHN_2021	China
MZ711360.1_	BCoV_ShX313_CHN_2021	China
OL456213.1_	HXD-4	China
OL960027.1_	BNS55	India

OL990398.1_	BFS-93	India
OP186313.1_	KGS-2	South_Korea
OP186314.1_	KTY-7	South_Korea
OP186315.1_	KJS-4	South_Korea
OP186316.1_	KBR-8	South_Korea
OP186317.1_	KJS-5	South_Korea
OP186318.1_	KBR-1	South_Korea
OP186319.1_	KBR-9	South_Korea
OP186320.1_	KHS	South_Korea
OP186321.1_	KJS-1	South_Korea
OP186322.1_	KBR-12	South_Korea
OP186323.1_	KSC-2	South_Korea
OP186324.1_	KNS-11	South_Korea
OP186325.1_	KBR-2	South_Korea
OP186326.1_	KIS-1	South_Korea
OP186327.1_	KHN-11	South_Korea
OP186328.1_	KHN-13	South_Korea
AF181469.1_	BCOV	unknown
M64667.1_	BCOV	unknown
M64668.1_	BCOV	unknown
D00662.1_	gene_for_S_peplomer_polypeptide_precursor	unknown
L07748.1_	Human_enteric_coronavirus_4408	unknown
MZ603735.1_	BCoV-S_CN_HB-SJZ_202	China
D00731.1_	Bovine_enteric_coronavirus	unknown
EF445634.1_	339_06e_(HE)_an	Italy
OP037398.1_	SDSU_2020_02_R	USA_California
OP037413.1_	SDSU_2022_04_R	USA_Nebraska
OP037409.1_	SDSU_2021_06_R	USA_North_Dakota
OP037412.1_	SDSU_2022_03_R	USA_Nebraska
OP037405.1_	SDSU_2021_02_R	USA_Nebraska
KX982264.1_	BCoV_2014_13	France
KX432213.1_	BJ232	China
OP037400.1_	SDSU_2020_04_R	USA_Wisconsin
OP037411.1_	SDSU_2022_02_R	USA_Nebraska
ON792957.1_	ABGEB0-128	Ireland
LC494190.1_	TCG-31_RNA	Japan
OP037404.1_	SDSU_2021_01_R	USA_Colorado
OP037410.1_	SDSU_2022_01_R	USA_Nebraska
LC494163.1_	TCG-2_RNA	Japan
LC494181.1_	TCG-22_RNA	Japan
ON792958.1_	ABGEBB-78	Ireland
LC494165.1_	TCG-10_RNA	Japan
LC494129.1_	IWT-1_RNA	Japan

LC494166.1_	TCG-11_RNA	Japan
LC494154.1_	SHG-1_RNA	Japan
LC494162.1_	TCG-3_RNA	Japan
LC494171.1_	TCG-16_RNA	Japan
LC494177.1_	TCG-9_RNA	Japan
LC494179.1_	TCG-20_RNA	Japan
LC494183.1_	TCG-24_RNA	Japan
LC494189.1_	TCG-30_RNA	Japan
LC494155.1_	SHG-2_RNA	Japan
LC494170.1_	TCG-15_RNA	Japan
LC494140.1_	IWT-14_RNA	Japan
LC494180.1_	TCG-21_RNA	Japan
MG757142.1_	ICSA-pool-LBA	France
FJ425185.1_	US_OH-WD358-GnC_1994	USA_Ohio
LC494161.1_	TCG-5_RNA	Japan
LC494182.1_	TCG-23_RNA	Japan
LC494184.1_	TCG-25_RNA	Japan
LC494187.1_	TCG-28_RNA	Japan
LC494136.1_	IWT-8_RNA	Japan
LC494175.1_	TCG-18_RNA	Japan
OP037406.1_	SDSU_2021_03_R	USA_Texas
LC494139.1_	IWT-13_RNA	Japan
LC494156.1_	SHG-3_RNA	Japan
LC494167.1_	TCG-12_RNA	Japan
LC494185.1_	TCG-26_RNA	Japan
LC494186.1_	TCG-27_RNA	Japan
LC494188.1_	TCG-29_RNA	Japan
LC494191.1_	TCG-32_RNA	Japan
LC494192.1_	TCG-33_RNA	Japan
LC494147.1_	IWT-17_RNA	Japan
LC494149.1_	IWT-23_RNA	Japan
LC494153.1_	IWT-27_RNA	Japan
LC494135.1_	IWT-7_RNA	Japan
LC494169.1_	TCG-14_RNA	Japan
LC494176.1_	TCG-17_RNA	Japan
LC494148.1_	IWT-22_RNA	Japan
LC494159.1_	SHG-6_RNA	Japan
LC494178.1_	TCG-19_RNA	Japan
LC494141.1_	IWT-15_RNA	Japan
LC494152.1_	IWT-26_RNA	Japan
LC494158.1_	SHG-5_RNA	Japan
LC494168.1_	TCG-13_RNA	Japan
LC494126.1_	GIF-1_RNA	Japan

LC494127.1_	IWT-9_RNA	Japan
LC494134.1_	IWT-6_RNA	Japan
LC494137.1_	IWT-11_RNA	Japan
LC494151.1_	IWT-25_RNA	Japan
LC494164.1_	TCG-1_RNA	Japan
LC494173.1_	TCG-7_RNA	Japan
LC494174.1_	TCG-8_RNA	Japan
LC494172.1_	TCG-6_RNA	Japan
FJ938066.1_	bovine_US_OH-440-TC_1996_complet	USA_Ohio
FJ938067.1_	Human_enteric_coronavirus_strain_4408	unknown
LC494157.1_	SHG-4_RNA	Japan
LC494128.1_	IWT-10_RNA	Japan
LC494130.1_	IWT-2_RNA	Japan
LC494132.1_	IWT-4_RNA	Japan
LC494133.1_	IWT-5_RNA	Japan
LC494138.1_	IWT-12_RNA	Japan
LC494142.1_	IWT-18_RNA	Japan
LC494143.1_	IWT-19_RNA	Japan
LC494144.1_	IWT-20_RNA	Japan
LC494145.1_	IWT-21_RNA	Japan
LC494146.1_	IWT-16_RNA	Japan
LC494150.1_	IWT-24_RNA	Japan
LC494160.1_	TCG-4_RNA	Japan
LC494131.1_	IWT-3_RNA	Japan
OP037403.1_	SDSU_2020_07_R	USA_Minnesota
ON792963.1_	ABGEDB-205	Ireland
FJ425186.1_	US_OH-WD358_1994	USA_Ohio
EF424620.1_	R-AH187	USA_Ohio
ON792944.1_	ABGEB0-59	Ireland
OP037407.1_	SDSU_2021_04_R	USA_Texas
FJ938065.1_	AH187	USA_Ohio
EF424616.1_	E-AH65-TC	USA_Ohio
KU886219.1_	BCV-AKS-01	China_Xinjiang
EF424622.1_	US_OH3-TC_2006	USA_Ohio
EF424624.1_	Calf-US_OH3_2006	USA_Ohio
FJ425190.1_	US_OH-WD388-TC_1994	USA_Ohio
OP037414.1_	VDC_2017_01_E	USA_Nebraska
OP037420.1_	VDC_2018_05_R	USA_Nebraska
OP037368.1_	MARC_2014_02_R	USA_Nebraska
ON093194.1_	BCoV2_2021_CHN	China
ON142317.1_	BCoV4_2021_CHN	China
EF424618.1_	R-AH65-TC	USA_Ohio
EF424619.1_	E-AH187	USA_Ohio

EF424621.1_	US_OH1_2003	USA_Ohio
FJ425184.1_	US_OH-WD358-TC_1994	USA_Ohio
FJ425188.1_	US_OH-WD388-TC_1994	USA_Ohio
FJ938064.1_	E-AH187-TC	USA_Ohio
OP037367.1_	MARC_2014_01_R	USA_Nebraska
OP037369.1_	MARC_2014_03_R	USA_Nebraska
OP037370.1_	MARC_2014_04_R	USA_Nebraska
FJ425189.1_	US_OH-WD388_1994	USA_Ohio
OP037387.1_	MARC_2018_02_R	USA_Nebraska
OP037437.1_	VDC_2022_02_E	USA_South_Dakota
EF424623.1_	US_OH3_2003	USA_Ohio
OP037397.1_	SDSU_2020_01_R	USA_California
OP037442.1_	VDC_2022_07_E	USA_Oregon
OP037378.1_	MARC_2016_06_R	USA_Nebraska
OP037389.1_	MARC_2019_01_R	USA_Nebraska
OP037377.1_	MARC_2016_05_R	USA_Nebraska
OP037383.1_	MARC_2017_04_E	USA_Nebraska
ON792962.1_	ABGEBB-129	Ireland
OP037374.1_	MARC_2016_02_R	USA_Nebraska
OP037384.1_	MARC_2017_05_R	USA_Nebraska
OP037390.1_	MARC_2019_02_R	USA_Nebraska
OP037394.1_	MARC_2021_01_R	USA_Nebraska
OP037419.1_	VDC_2018_04_R	USA_Nebraska
OP037376.1_	MARC_2016_04_R	USA_Nebraska
OP037379.1_	MARC_2016_07_R	USA_Nebraska
OP037416.1_	VDC_2018_01_R	USA_Nebraska
OP037428.1_	VDC_2018_13_E	USA_Nebraska
OP037441.1_	VDC_2022_06_E	USA_Nebraska
DQ811784.2_	DB2	unknown
OP037365.1_	MARC_2013_01_R	USA_Nebraska
OP037395.1_	MARC_2021_02_R	USA_Nebraska
OP037402.1_	SDSU_2020_06_R	USA_Nebraska
OP037418.1_	VDC_2018_03_R	USA_Nebraska
OP037433.1_	VDC_2019_04_E	USA_Nebraska
MW711287.1_	SWUN_NMG-D10_2020	China
ON142315.1_	BCoV1_2021_CHN	China
ON142320.1_	BCoV7_2021_CHN	China
OP037382.1_	MARC_2017_03_E	USA_Nebraska
OP037396.1_	MARC_2021_03_R	USA_Nebraska
OP037417.1_	VDC_2018_02_R	USA_Wyoming
OP037424.1_	VDC_2018_09_E	USA_Nebraska
OP037427.1_	VDC_2018_12_E	USA_Nebraska
OP037432.1_	VDC_2019_03_E	USA_Nebraska

OP037435.1_	VDC_2019_06_E	USA_Nebraska
ON142318.1_	UNVERIFIED_BCoV5_2021_CHN_complet	China
OP037373.1_	MARC_2016_01_R	USA_Nebraska
OP037375.1_	MARC_2016_03_R	USA_Nebraska
OP037381.1_	MARC_2017_02_E	USA_Nebraska
OP037415.1_	VDC_2017_02_EN	USA_Nebraska
ON142316.1_	BCoV3_2021_CHN	China
OP037391.1_	MARC_2020_01_R	USA_Nebraska
OP037439.1_	VDC_2022_04_E	USA_Nebraska
OP037421.1_	VDC_2018_06_E	USA_Nebraska
OP037429.1_	VDC_2019_01_E	USA_Nebraska
OP037425.1_	VDC_2018_10_E	USA_Nebraska
OP037380.1_	MARC_2017_01_R	USA_Nebraska
OP037392.1_	MARC_2020_02_R	USA_Nebraska
OP037386.1_	MARC_2018_01_R	USA_Nebraska
OP037423.1_	VDC_2018_08_E	USA_Pennsylvania
OP037426.1_	VDC_2018_11_E	USA_Nebraska
OP037430.1_	VDC_2019_01_R	USA_Nebraska
LC642814.1_	BoCoV_GF2020_RNA	Japan
OP037434.1_	VDC_2019_05_E	USA_Nebraska
EF424617.1_	R-AH65	USA_Ohio
MH043954.1_	4-17-08	USA_Pennsylvania
ON146444.1_	MARC_BRCV_2014	USA_Nebraska
OP037366.1_	MARC_2013_02_R	USA_Nebraska
OP037372.1_	MARC_2015_02_R	USA_Nebraska
OP037388.1_	MARC_2018_05_E	USA_Nebraska
OP037401.1_	SDSU_2020_05_R	USA_Minnesota
OP037422.1_	VDC_2018_07_E	USA_Nebraska
OP037431.1_	VDC_2019_02_E	USA_Nebraska
OP037438.1_	VDC_2022_03_E	USA_South_Dakota
EF424615.1_	E-AH65	USA_Ohio
ON792950.1_	ABGEB0-78	Ireland
OP037371.1_	MARC_2015_01_R	USA_Nebraska
OP037385.1_	MARC_2017_06_R	USA_Nebraska
MN982198.1_	BCOV-China_SWUN_A1_2018	China
MG757141.1_	ICSA-pool-EN	France
MH043952.1_	4-17-03	USA_Pennsylvania
OP037408.1_	SDSU_2021_05_R	USA_California
LQ289146.1_	Sequence_25_from_Patent_WO2016034610	unknown
OP037436.1_	VDC_2022_01_E	USA_Kansas
FJ938063.1_	E-DB2-TC	USA_Ohio
MG757140.1_	ICSA16-LBA	France
MG757138.1_	ICSA21-LBA	France

OP037399.1_	SDSU_2020_03_R	USA_Texas
AF391541.1_	BCoV-ENT	unknown
AF391542.1_	BCoV-LUN	unknown
MG757139.1_	ICSA16-EN	France
MN982199.1_	BCOV-China_SWUN_A10_2018	China
MO401628.1_	Sequence_25_from_patent_US_10434168	unknown
FJ415324.1_	Human_enteric_coronavirus_4408	Germany
MH043953.1_	4-17-25	USA_Pennsylvania
MH043955.1_	7-16-23	USA_Pennsylvania
MH810163.1_	Yak_coronavirus_strain_YAK_HY24_CH_2017	China
ON792943.1_	ABGEB0-56	Ireland
OP296992.1_	p95	Taiwan
U00735.2_	Mebus	unknown
OP037440.1_	VDC_2022_05_E	USA_Nebraska
MG518518.1_	W17-18	South_Korea_Chungnam
ON792964.1_	ABGEDB-260	Ireland
ON792959.1_	ABGEBB-108	Ireland
OP037393.1_	MARC_2020_03_R	USA_Nebraska
AB354579.1_	genomic_RNA_strain_Kakegawa	Japan
ON792942.1_	ABGEB0-54	Ireland
ON142319.1_	BCoV6_2021_CHN	China
ON792956.1_	ABGEB0-122	Ireland
ON792954.1_	ABGEB0-110	Ireland
ON792951.1_	ABGEB0-79	Ireland
ON792947.1_	ABGEB0-70	Ireland
ON792953.1_	ABGEB0-88	Ireland
DQ915164.2_	Alpaca	USA
ON792949.1_	ABGEB0-74	Ireland
ON792946.1_	ABGEB0-66	Ireland
AF220295.1_	Quebec	unknown
ON792955.1_	ABGEB0-116	Ireland
ON792948.1_	ABGEB0-72	Ireland
ON792960.1_	ABGEBB-110	Ireland
ON792961.1_	ABGEBB-115	Ireland
ON792952.1_	ABGEB0-81	Ireland
ON792941.1_	ABGEB0-52	Ireland
ON792945.1_	ABGEB0-62	Ireland

HE gene sequences (371 sequences)

Accession number	Strain name	Country/Region
OR676674.1_	Ovine/SWUN/FA/2023	China
OR676675.1_	Ovine/SWUN/28-3/2023	China
OR603986.1_	Yak/SWUN/NF2/2022	China
OR603987.1_	Yak/SWUN/NF5/2022	China
OR612023.1_	BCoV/SWUN/HXD2/2022	China
OR612024.1_	BCoV/SWUN/HXD3/2022	China
OR612025.1_	BCoV/SWUN/HXD4/2022	China
OR612026.1_	BCoV/SWUN/HXD6/2022	China
OR612027.1_	BCoV/SWUN/HXD7/2022	China
OR621177.1_	BCoV/SWUN/XHD3/2022	China
OR621176.1_	BCoV/SWUN/XHD1/2022	China
OR621178.1_	BCoV/SWUN/XHD4/2022	China
OR621175.1_	BCoV/SWUN/HXD8/2022	China
OR603988.1_	BCoV/SWUN/HXD9/2022	China
OR612022.1_	BCoV/SWUN/HXD1/2022	China
OR612021.1_	BCoV/SWUN/XHD5/2022	China
OR612020.1_	BCoV/SWUN/XHD6/2022	China
OR621174.1_	BCoV/SWUN/XHD7/2022	China
OR603989.1_	BCoV/SWUN/HXD5/2022	China
OR612019.1_	BCoV/SWUN/HXD10/2022	China
OR596700.1_	BCoV/SWUN/XHD2/2022	China
OR077310.1_	YC2304G	China
OR077311.1_	XJCJ2301G	China
OR454204.1_	BCoV/YNZT/2023	China
OR077317.1_	ZJNB2106	China
OR077308.1_	RG23F3	China
OR077316.1_	JSYZ2209	China
OR077315.1_	HBSJZ2202	China
OR750853.1_	BCoV-XJ-SHZ-37	China
OR077313.1_	GZBJ2207	China
OR077312.1_	HBLF2302	China
OR077314.1_	HBSJZ2112	China
OP037419.1_	VDC/2018/04/R	USA
OR753439.1_	BCo/YNLP/2023	China
OR077306.1_	HMsz2207	China
OR077307.1_	ZJ2303G	China
OR088593.1_	BCoV/YN1LC/2023	China
OR077309.1_	AHFY2302G	China
OR947456.1_	BCoV/NX-1/CHN/2022	China
OP004056.2_	CH-4-22-01	USA
DQ915164.2_	Alpaca(2)	USA

MG518518.1_	Water_deer_W17-18	Korea
FJ425187.1_	White-tailed_deer_US/OH-WD470/1994	USA
EF424623.1_	Giraffe_coronavirus_US/OH3/2003	USA
FJ425189.1_	Sambar_deer_coronavirus_US/OH-WD388/1994	USA
EF424621.1_	Sable_antelope_coronavirus_US/OH1/2003	USA
FJ425184.1_	Waterbuck_coronavirus_US/OH-WD358-TC/1994	USA
FJ425188.1_	Sambar_deer_coronavirus_US/OH-WD388-TC/1994	USA
ON014593.1_	Bovine-like_Dama_M0032	UK
FJ425190.1_	Sambar_deer_US/OH-WD388-TC/1994	USA
EF424622.1_	Giraffe_US/OH3-TC/2006	USA
EF424624.1_	Calf-giraffe_US/OH3/2006	USA
FJ425186.1_	Waterbuck_US/OH-WD358/1994	USA
FJ425185.1_	Waterbuck_US/OH-WD358-GnC/1994	USA
MZ100070.1_	Tapir_coronavirus_1044512-1	USA
ON792945.1_	ABGEB0-62	Ireland
ON792941.1_	ABGEB0-52	Ireland
ON792952.1_	ABGEB0-81	Ireland
ON792960.1_	ABGEBB-110	Ireland
ON792961.1_	ABGEBB-115	Ireland
ON792948.1_	ABGEB0-72	Ireland
ON792955.1_	ABGEB0-116	Ireland
AF220295.1_	Quebec	unknown
ON792946.1_	ABGEB0-66	Ireland
ON792949.1_	ABGEB0-74	Ireland
DQ915164.2_	Alpaca	USA
ON792947.1_	ABGEB0-70	Ireland
ON792953.1_	ABGEB0-88	Ireland
ON792951.1_	ABGEB0-79	Ireland
ON792954.1_	ABGEB0-110	Ireland
ON792956.1_	ABGEB0-122	Ireland
ON142319.1_	BCoV6_2021_CHN	China
ON792942.1_	ABGEB0-54	Ireland
AB354579.1_	Kakegawa	Japan
OP037393.1_	MARC_2020_03_R	USA_Nebraska
ON792959.1_	ABGEBB-108	Ireland
MG518518.1_	W17-18	South_Korea_Chungnam
ON792964.1_	ABGEDB-260	Ireland
OP037440.1_	VDC_2022_05_E	USA_Nebraska
U00735.2_	Mebus	unknown
MH043953.1_	4-17-25	USA_Pennsylvania
MH043955.1_	7-16-23	USA_Pennsylvania
MH810163.1_	YAK_HY24_CH_2017	China
ON792943.1_	ABGEB0-56	Ireland

OP296992.1_	p95	Taiwan
FJ415324.1_	Human_enteric_coronavirus_4408	Germany
AF391541.1_	BCoV-ENT	unknown
AF391542.1_	BCoV-LUN	unknown
MG757139.1_	ICSA16-EN	France
MO401628.1_	10434168	unknown
MN982199.1_	BCOV-China_SWUN_A10_2018	China
OP037399.1_	SDSU_2020_03_R	USA_Texas
MG757138.1_	ICSA21-LBA	France
FJ938063.1_	E-DB2-TC	USA_Ohio
MG757140.1_	ICSA16-LBA	France
OP037436.1_	VDC_2022_01_E	USA_Kansas
LQ289146.1_	WO2016034610	unknown
OP037408.1_	SDSU_2021_05_R	USA_California
FJ425187.1_	US_OH-WD470_1994	USA_Ohio
MG757141.1_	ICSA-pool-EN	France
MH043952.1_	4-17-03	USA_Pennsylvania
MN982198.1_	BCOV-China_SWUN_A1_2018	China
EF424615.1_	E-AH65	USA_Ohio
ON792950.1_	ABGEB0-78	Ireland
OP037371.1_	MARC_2015_01_R	USA_Nebraska
OP037385.1_	MARC_2017_06_R	USA_Nebraska
EF424617.1_	R-AH65	USA_Ohio
MH043954.1_	4-17-08	USA_Pennsylvania
ON146444.1_	MARC_BRCV_2014	USA_Nebraska
OP037366.1_	MARC_2013_02_R	USA_Nebraska
OP037372.1_	MARC_2015_02_R	USA_Nebraska
OP037388.1_	MARC_2018_05_E	USA_Nebraska
OP037401.1_	SDSU_2020_05_R	USA_Minnesota
OP037422.1_	VDC_2018_07_E	USA_Nebraska
OP037431.1_	VDC_2019_02_E	USA_Nebraska
OP037438.1_	VDC_2022_03_E	USA_South_Dakota
LC642814.1_	BoCoV_GF2020_RNA	Japan
OP037434.1_	VDC_2019_05_E	USA_Nebraska
OP037386.1_	MARC_2018_01_R	USA_Nebraska
OP037423.1_	VDC_2018_08_E	USA_Pennsylvania
OP037426.1_	VDC_2018_11_E	USA_Nebraska
OP037430.1_	VDC_2019_01_R	USA_Nebraska
OP037380.1_	MARC_2017_01_R	USA_Nebraska
OP037392.1_	MARC_2020_02_R	USA_Nebraska
OP037425.1_	VDC_2018_10_E	USA_Nebraska
OP037421.1_	VDC_2018_06_E	USA_Nebraska
OP037429.1_	VDC_2019_01_E	USA_Nebraska

OP037391.1_	MARC_2020_01_R	USA_Nebraska
OP037439.1_	VDC_2022_04_E	USA_Nebraska
ON142316.1_	BCoV3_2021_CHN	China
OP037373.1_	MARC_2016_01_R	USA_Nebraska
OP037375.1_	MARC_2016_03_R	USA_Nebraska
OP037381.1_	MARC_2017_02_E	USA_Nebraska
OP037415.1_	VDC_2017_02_EN	USA_Nebraska
ON142318.1_	UNVERIFIED_BCoV5_2021_CHN	China
MW711287.1_	SWUN_NMG-D10_2020	China
OP037382.1_	MARC_2017_03_E	USA_Nebraska
OP037396.1_	MARC_2021_03_R	USA_Nebraska
OP037417.1_	VDC_2018_02_R	USA_Wyoming
OP037424.1_	VDC_2018_09_E	USA_Nebraska
OP037427.1_	VDC_2018_12_E	USA_Nebraska
OP037432.1_	VDC_2019_03_E	USA_Nebraska
OP037435.1_	VDC_2019_06_E	USA_Nebraska
ON142315.1_	BCoV1_2021_CHN	China
ON142320.1_	BCoV7_2021_CHN	China
DQ811784.2_	DB2	unknown
OP037365.1_	MARC_2013_01_R	USA_Nebraska
OP037395.1_	MARC_2021_02_R	USA_Nebraska
OP037402.1_	SDSU_2020_06_R	USA_Nebraska
OP037418.1_	VDC_2018_03_R	USA_Nebraska
OP037433.1_	VDC_2019_04_E	USA_Nebraska
OP037376.1_	MARC_2016_04_R	USA_Nebraska
OP037379.1_	MARC_2016_07_R	USA_Nebraska
OP037416.1_	VDC_2018_01_R	USA_Nebraska
OP037428.1_	VDC_2018_13_E	USA_Nebraska
OP037441.1_	VDC_2022_06_E	USA_Nebraska
ON792962.1_	ABGEBB-129	Ireland
OP037374.1_	MARC_2016_02_R	USA_Nebraska
OP037384.1_	MARC_2017_05_R	USA_Nebraska
OP037390.1_	MARC_2019_02_R	USA_Nebraska
OP037394.1_	MARC_2021_01_R	USA_Nebraska
OP037419.1_	VDC_2018_04_R	USA_Nebraska
OP037377.1_	MARC_2016_05_R	USA_Nebraska
OP037383.1_	MARC_2017_04_E	USA_Nebraska
OP037378.1_	MARC_2016_06_R	USA_Nebraska
OP037389.1_	MARC_2019_01_R	USA_Nebraska
EF424623.1_	US_OH3_2003	USA_Ohio
OP037397.1_	SDSU_2020_01_R	USA_California
OP037442.1_	VDC_2022_07_E	USA_Oregon
OP037437.1_	VDC_2022_02_E	USA_South_Dakota

OP037387.1_	MARC_2018_02_R	USA_Nebraska
FJ425189.1_	US_OH-WD388_1994	USA_Ohio
OP037367.1_	MARC_2014_01_R	USA_Nebraska
OP037369.1_	MARC_2014_03_R	USA_Nebraska
OP037370.1_	MARC_2014_04_R	USA_Nebraska
EF424618.1_	R-AH65-TC	USA_Ohio
EF424619.1_	E-AH187	USA_Ohio
EF424621.1_	US_OH1_2003	USA_Ohio
FJ425184.1_	US_OH-WD358-TC_1994	USA_Ohio
FJ425188.1_	US_OH-WD388-TC_1994	USA_Ohio
FJ938064.1_	E-AH187-TC	USA_Ohio
ON142317.1_	BCoV4_2021_CHN	China
ON093194.1_	BCoV2_2021_CHN	China
OP037368.1_	MARC_2014_02_R	USA_Nebraska
OP037420.1_	VDC_2018_05_R	USA_Nebraska
OP037414.1_	VDC_2017_01_E	USA_Nebraska
FJ425190.1_	US_OH-WD388-TC_1994	USA_Ohio
EF424622.1_	US_OH3-TC_2006	USA_Ohio
EF424624.1_	US_OH3_2006	USA_Ohio
KU886219.1_	BCV-AKS-01	China_Xinjiang
EF424616.1_	E-AH65-TC	USA_Ohio
FJ938065.1_	AH187	USA_Ohio
ON792944.1_	ABGEB0-59	Ireland
OP037407.1_	SDSU_2021_04_R	USA_Texas
EF424620.1_	R-AH187	USA_Ohio
FJ425186.1_	US_OH-WD358_1994	USA_Ohio
ON792963.1_	ABGEDB-205	Ireland
OP037403.1_	SDSU_2020_07_R	USA_Minnesota
LC494131.1_	IWT-3_RNA	Japan
LC494128.1_	IWT-10_RNA	Japan
LC494130.1_	IWT-2_RNA	Japan
LC494132.1_	IWT-4_RNA	Japan
LC494133.1_	IWT-5_RNA	Japan
LC494138.1_	IWT-12_RNA	Japan
LC494142.1_	IWT-18_RNA	Japan
LC494143.1_	IWT-19_RNA	Japan
LC494144.1_	IWT-20_RNA	Japan
LC494145.1_	IWT-21_RNA	Japan
LC494146.1_	IWT-16_RNA	Japan
LC494150.1_	IWT-24_RNA	Japan
LC494160.1_	TCG-4_RNA	Japan
FJ938066.1_	US_OH-440-TC_1996	USA_Ohio
FJ938067.1_	Human_enteric_coronavirus_strain_4408	unknown

LC494157.1_	SHG-4_RNA	Japan
LC494172.1_	TCG-6_RNA	Japan
LC494126.1_	GIF-1_RNA	Japan
LC494127.1_	IWT-9_RNA	Japan
LC494134.1_	IWT-6_RNA	Japan
LC494137.1_	IWT-11_RNA	Japan
LC494151.1_	IWT-25_RNA	Japan
LC494164.1_	TCG-1_RNA	Japan
LC494173.1_	TCG-7_RNA	Japan
LC494174.1_	TCG-8_RNA	Japan
LC494141.1_	IWT-15_RNA	Japan
LC494152.1_	IWT-26_RNA	Japan
LC494158.1_	SHG-5_RNA	Japan
LC494168.1_	TCG-13_RNA	Japan
LC494178.1_	TCG-19_RNA	Japan
LC494159.1_	SHG-6_RNA	Japan
LC494148.1_	IWT-22_RNA	Japan
LC494135.1_	IWT-7_RNA	Japan
LC494169.1_	TCG-14_RNA	Japan
LC494176.1_	TCG-17_RNA	Japan
LC494147.1_	IWT-17_RNA	Japan
LC494149.1_	IWT-23_RNA	Japan
LC494153.1_	IWT-27_RNA	Japan
LC494185.1_	TCG-26_RNA	Japan
LC494186.1_	TCG-27_RNA	Japan
LC494188.1_	TCG-29_RNA	Japan
LC494191.1_	TCG-32_RNA	Japan
LC494192.1_	TCG-33_RNA	Japan
LC494139.1_	IWT-13_RNA	Japan
LC494156.1_	SHG-3_RNA	Japan
LC494167.1_	TCG-12_RNA	Japan
LC494136.1_	IWT-8_RNA	Japan
LC494175.1_	TCG-18_RNA	Japan
OP037406.1_	SDSU_2021_03_R	USA_Texas
FJ425185.1_	US_OH-WD358-GnC_1994	USA_Ohio
LC494182.1_	TCG-23_RNA	Japan
LC494184.1_	TCG-25_RNA	Japan
LC494187.1_	TCG-28_RNA	Japan
LC494161.1_	TCG-5_RNA	Japan
MG757142.1_	ICSA-pool-LBA	France
LC494180.1_	TCG-21_RNA	Japan
LC494140.1_	IWT-14_RNA	Japan
LC494155.1_	SHG-2_RNA	Japan

LC494170.1_	TCG-15_RNA	Japan
LC494189.1_	TCG-30_RNA	Japan
LC494179.1_	TCG-20_RNA	Japan
LC494183.1_	TCG-24_RNA	Japan
LC494154.1_	SHG-1_RNA	Japan
LC494162.1_	TCG-3_RNA	Japan
LC494171.1_	TCG-16_RNA	Japan
LC494177.1_	TCG-9_RNA	Japan
LC494129.1_	IWT-1_RNA	Japan
LC494166.1_	TCG-11_RNA	Japan
LC494165.1_	TCG-10_RNA	Japan
ON792958.1_	ABGEBB-78	Ireland
LC494181.1_	TCG-22_RNA	Japan
LC494163.1_	TCG-2_RNA	Japan
OP037410.1_	SDSU_2022_01_R	USA_Nebraska
OP037404.1_	SDSU_2021_01_R	USA_Colorado
OP037411.1_	SDSU_2022_02_R	USA_Nebraska
OP037400.1_	SDSU_2020_04_R	USA_Wisconsin
OP037405.1_	SDSU_2021_02_R	USA_Nebraska
OP037412.1_	SDSU_2022_03_R	USA_Nebraska
OP037413.1_	SDSU_2022_04_R	USA_Nebraska
OP037398.1_	SDSU_2020_02_R	USA_California
M76372.1_	L9	unknown
M84486.1_	LY138	unknown
ON792957.1_	ABGEB0-128	Ireland
AF230523.1_	Quebec_BCQ.2508	unknown
OP037409.1_	SDSU_2021_06_R	USA_North_Dakota
KX432213.1_	BJ232	China
KX982264.1_	BCoV_2014_13	France
AF230524.1_	Quebec_BCQ.2442	unknown
AF230525.1_	Quebec_BCQ.2439	unknown
LC494190.1_	TCG-31_RNA	Japan
AF230526.1_	Quebec_BCQ.376	unknown
AF230527.1_	Quebec_BCQ.701	unknown
AF230528.1_	Quebec_BCQ.3708	unknown
L07747.1_	Human_enteric_coronavirus_4408	unknown
OP762501.1_	BCoV_China_cattle_F273_2021	China
OP762502.1_	BCoV_China_cattle_B299_2021	China
OP762503.1_	BCoV_China_cattle_B298_2021	China
DQ389642.1_	KCD1	South_Korea
DQ389643.1_	KCD2	South_Korea
DQ389644.1_	KCD3	South_Korea
DQ389645.1_	KCD4	South_Korea

DQ389646.1_	KCD5	South_Korea
DQ389647.1_	KCD6	South_Korea
DQ389648.1_	KCD7	South_Korea
DQ389649.1_	KCD8	South_Korea
DQ389650.1_	KCD9	South_Korea
DQ389651.1_	KCD10	South_Korea
DQ994162.1_	KWD11	South_Korea
DQ994163.1_	KWD12	South_Korea
DQ994164.1_	KWD13	South_Korea
DQ994165.1_	KWD14	South_Korea
DQ994166.1_	KWD15	South_Korea
DQ994167.1_	KWD16	South_Korea
DQ994168.1_	KWD17	South_Korea
DQ994169.1_	KWD18	South_Korea
DQ994170.1_	KWD19	South_Korea
EU401975.1_	501	South_Korea
EU401976.1_	502	South_Korea
EU401977.1_	A3	South_Korea
EU401978.1_	SUN5	South_Korea
EU401979.1_	BC94	South_Korea
HM573321.1_	Wisent_10_01	South_Korea
HM573322.1_	Himalayan_Tahr1_10_0	South_Korea
HM573323.1_	Himalayan_Tahr2_10_0	South_Korea
HM573324.1_	Sitatunga_10_01	South_Korea
HM573325.1_	Nyala_10_01	South_Korea
KM985632.1_	HLJ-14_CHN	China
MH203059.1_	ND65	Viet_Nam
MH203060.1_	PL83	Viet_Nam
MH203061.1_	PL84	Viet_Nam
MH203062.1_	DT97	Viet_Nam
MH203063.1_	BL104	Viet_Nam
MK095135.1_	BCOV-China_SWUN_SC1_201	China
MK095136.1_	BCOV-China_SWUN_SC2_201	China
MK095137.1_	BCOV-China_SWUN_SC3_201	China
MK095138.1_	BCOV-China_SWUN_SX1_201	China
MK095139.1_	BCOV-China_SWUN_SX2_201	China
MK095140.1_	BCOV-China_SWUN_LN1_201	China
MK095141.1_	BCOV-China_SWUN_LN2_201	China
MK095142.1_	BCOV-China_SWUN_LN3_201	China
MK095143.1_	BCOV-China_SWUN_LN4_201	China
MK095144.1_	BCOV-China_SWUN_LN5_201	China
MK095145.1_	BCOV-China_SWUN_HN1_201	China
MK095146.1_	BCOV-China_SWUN_HN2_201	China

MK095147.1_	BCOV-China_SWUN_HN3_201	China
MK903507.1_	BCoV_CH_HB-BD_201	China
OF134845.1_	KR_1020210038171-A_23	unknown
MZ825078.1_	KCD_GJ2_2019	South_Korea
MZ825082.1_	KCD_GJ2_2020	South_Korea
MZ825083.1_	KCD_BA_2020	South_Korea
OP186329.1_	KGS-2	South_Korea
OP186330.1_	KTY-7	South_Korea
OP186331.1_	KJS-4	South_Korea
OP186332.1_	KBR-8	South_Korea
OP186333.1_	KJS-5	South_Korea
OP186334.1_	KBR-1	South_Korea
OP186335.1_	KBR-9	South_Korea
OP186336.1_	KHS	South_Korea
OP186337.1_	KJS-1	South_Korea
OP186338.1_	KBR-12	South_Korea
OP186339.1_	KSC-2	South_Korea
OP186340.1_	KNS-11	South_Korea
OP186341.1_	KBR-2	South_Korea
OP186342.1_	KIS-1	South_Korea
OP186343.1_	KHN-11	South_Korea
OP186344.1_	KHN-13	South_Korea
OP762504.1_	BCoV_China_cattle_F226_2021	China
OP762505.1_	BCoV_China_cattle_B277a_2021	China
OP762507.1_	BCoV_China_cattle_B259_2021	China
OP762508.1_	BCoV_China_cattle_B236_2021	China
OP762509.1_	BCoV_China_cattle_B229_2021	China
OP762510.1_	BCoV_China_cattle_B226_2021	China
OP762511.1_	BCoV_China_cattle_B224_2021	China

Supplementary Table S4. Individual Ct values of BCoV RNA detected in sheep samples.

Fecal samples from diseased sheep														
**NO.**	**F1**	**F2**	**F3**	**F4**	**F5**	**F6**	**F7**	**F8**	**F9**	**F10**	**F11**	**F12**	**F13**	**F14**
**Ct**	**29.58**	**29.79**	**ND**	**28.16**	**29.24**	**31.19**	**31.78**	**30.36**	**33.12**	**29.13**	**32.09**	**33.12**	**30.65**	**29.99**
**Fecal samples from clinically healthy sheep**														
**NO.**	**1**	**2**	**3**	**4**	**5**	**6**	**7**	**8**	**9**	**10**	**11**	**12**		
**Ct**	**32.53**	**ND**	**ND**	**ND**	**ND**	**ND**	**33.91**	**ND**	**32.07**	**ND**	**37.52**	**34.19**		
**NO.**	**13**	**14**	**15**	**16**	**17**	**18**	**19**	**20**	**21**	**22**	**23**	**24**		
**Ct**	**35.66**	**ND**	**ND**	**37.44**	**ND**	**35.69**	**ND**	**37.93**	**ND**	**34.44**	**31.17**	**ND**		
**NO.**	**25**	**26**	**27**	**28**	**29**	**30**	**31**	**32**	**33**	**34**	**35**	**36**		
**Ct**	**ND**	**33.08**	**30.92**	**33.17**	**ND**	**33.53**	**31.25**	**ND**	**38.65**	**ND**	**ND**	**33.08**		
**NO.**	**37**	**38**	**39**	**40**	**41**									
**Ct**	**ND**	**31.15**	**ND**	**ND**	**39.15**									

Note: Ct values were obtained by RT-qPCR detection of BCoV RNA.

Supplementary Table S5. Viral shedding detection in experimentally infected sheep

Animal ID	Sample type	Pre-infection	1 DPI	2 DPI	3 DPI	4 DPI	5 DPI
**Sheep 1**	**Nasal swab**	**ND**	**ND**	**ND**	**36.76**	**32.05**	**35.45**
**Sheep 1**	**Feces**	**ND**	**ND**	**39.46**	**37.06**	**33.63**	**32.86**
**Sheep 1**	**Serum**	**ND**	**ND**	**ND**	**ND**	**33.76**	**35.62**
**Sheep 2**	**Nasal swab**	**ND**	**35.38**	**35.73**	**35.70**	**33.84**	**ND**
**Sheep 2**	**Feces**	**ND**	**37.46**	**37.14**	**35.47**	**31.52**	**ND**
**Sheep 2**	**Serum**	**ND**	**ND**	**ND**	**ND**	**35.22**	**ND**
**Sheep 3**	**Nasal swab**	**ND**	**37.16**	**37.57**	**35.37**	**33.42**	**34.21**
**Sheep 3**	**Feces**	**ND**	**38.69**	**37.77**	**35.09**	**31.93**	**32.93**
**Sheep 3**	**Serum**	**ND**	**ND**	**ND**	**ND**	**33.86**	**34.50**
**Negative control 1**	**Nasal swab**	**ND**	**ND**	**ND**	**ND**	**ND**	**ND**
**Negative control 1**	**Feces**	**ND**	**ND**	**ND**	**ND**	**ND**	**ND**
**Negative control 1**	**Serum**	**ND**	**ND**	**ND**	**ND**	**ND**	**ND**
**Negative control 2**	**Nasal swab**	**ND**	**ND**	**ND**	**ND**	**ND**	**ND**
**Negative control 2**	**Feces**	**ND**	**ND**	**ND**	**ND**	**ND**	**ND**
**Negative control 2**	**Serum**	**ND**	**ND**	**ND**	**ND**	**ND**	**ND**

Note: Values are presented as mean Ct values from three technical replicates. ND, not detected.
